# A clue to unprecedented strategy to HIV eradication: “Lock-in and apoptosis”

**DOI:** 10.1038/s41598-017-09129-w

**Published:** 2017-08-21

**Authors:** Hiroshi Tateishi, Kazuaki Monde, Kensaku Anraku, Ryoko Koga, Yuya Hayashi, Halil Ibrahim Ciftci, Hasan DeMirci, Taishi Higashi, Keiichi Motoyama, Hidetoshi Arima, Masami Otsuka, Mikako Fujita

**Affiliations:** 1 Department of Bioorganic Medicinal Chemistry, Faculty of Life Sciences, Kumamoto University, Kumamoto, Japan; 2 Department of Microbiology, Faculty of Life Sciences, Kumamoto University, Kumamoto, Japan; 3 Department of Medical Technology, Kumamoto Health Science University, Kumamoto, Japan; 4 Department of Physical Pharmaceutics, Faculty of Life Sciences, Kumamoto University, Kumamoto, Japan; 5 Stanford PULSE Institute, SLAC National Accelerator Laboratory, Menlo Park, California, USA; 6 Biosciences Division, SLAC National Accelerator Laboratory, Menlo Park, California, USA; 7 Research Institute for Drug Discovery, School of Pharmacy, Kumamoto University, Kumamoto, Japan

## Abstract

Despite the development of antiretroviral therapy against HIV, eradication of the virus from the body, as a means to a cure, remains in progress. A “kick and kill” strategy proposes “kick” of the latent HIV to an active HIV to eventually be “killed”. Latency-reverting agents that can perform the “kick” function are under development and have shown promise. Management of the infected cells not to produce virions after the “kick” step is important to this strategy. Here we show that a newly synthesized compound, L-HIPPO, captures the HIV-1 protein Pr55^Gag^ and intercepts its function to translocate the virus from the cytoplasm to the plasma membrane leading to virion budding. The infecting virus thus “locked-in” subsequently induces apoptosis of the host cells. This “lock-in and apoptosis” approach performed by our novel compound in HIV-infected cells provides a means to bridge the gap between the “kick” and “kill” steps of this eradication strategy. By building upon previous progress in latency reverting agents, our compound appears to provide a promising step toward the goal of HIV eradication from the body.

## Introduction

Although human immunodeficiency virus (HIV)/acquired immunodeficiency syndrome (AIDS) has become amenable to control since 1997 by antiretroviral therapy (ART), HIV remains incurable to date^1^. In 2015, approximately 36.7 million people were living with HIV and 1.1 million people died of AIDS^2^. Most anti-HIV drugs work by blocking key steps of the viral replication cycle, making them effective against actively-replicating viruses, but have little effect on latent HIV-1 in that remains in cellular reservoirs throughout the body. The viral replication cycle is essentially shut down in the latent virus, allowing it to persist in the body even with the administration of comprehensive ART. A cure for HIV would mean the lack of viral activity after treatment which is not the case with current ART. Such strategies toward this end include the goal of eliminating the cellular reservoirs which continue to harbor HIV^3^. Previously, a “kick and kill” strategy was proposed to connect a “kick” process to a subsequent “kill” process for HIV-infected cells, wherein “kicked,” i.e. cells containing active HIV, can be placed in fatal crisis by such means as a heightened immune response or the induction of cell damage^4^. The discovery of latency reverting agents (LRA) that “kick” the latent virus-containing cells to activate the expression of HIV-1 protein has signified promising progress toward this approach^4^. However, efficient “kill” device remains to be developed^4, 5^. We report herein a novel “lock-in and apoptosis” compound that follows the “kick” process and facilitates eradication of HIV-infected cells.

**Figure 1:**
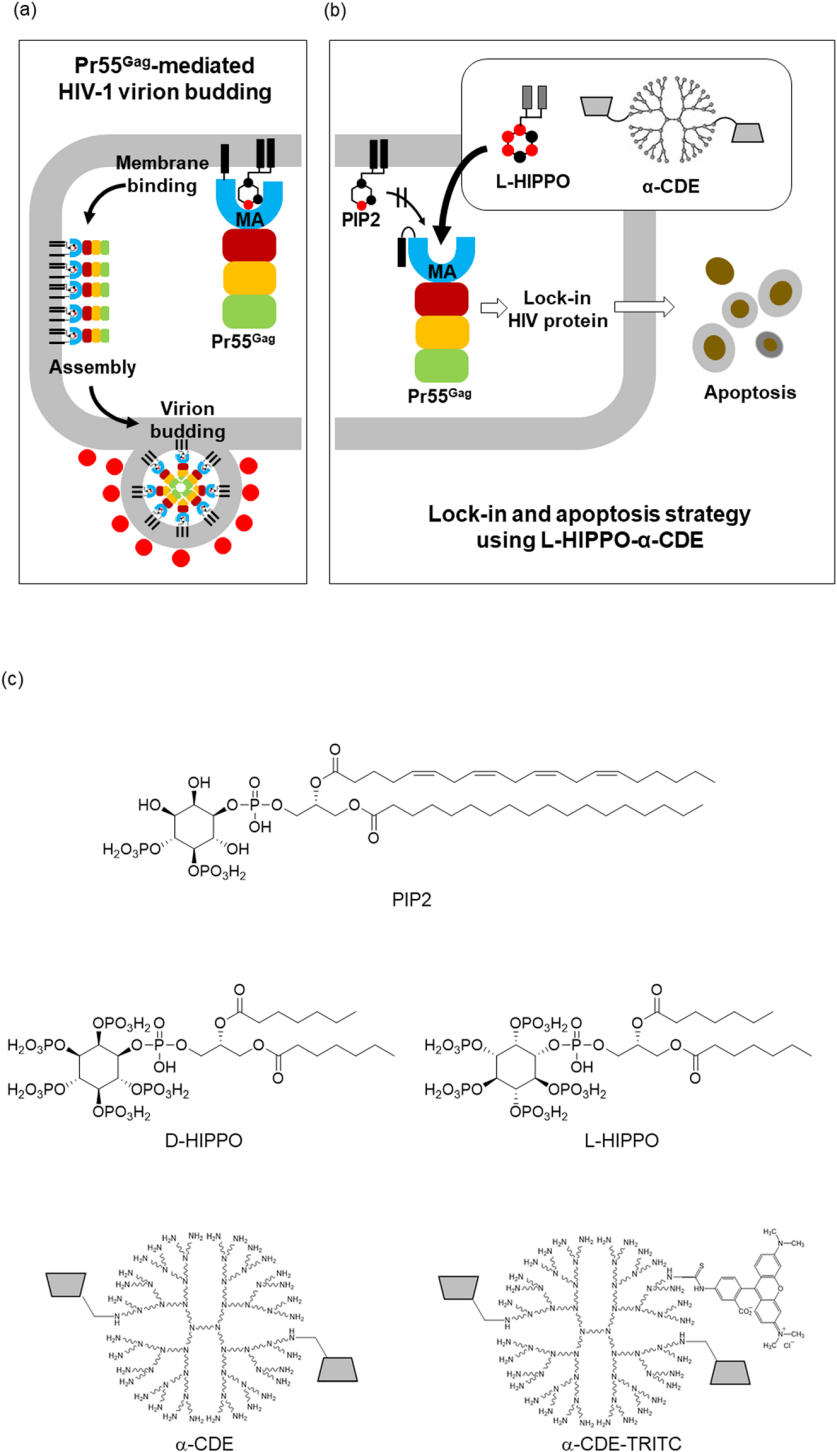
Lock-in and apoptosis strategy using a cyclodextrin/dendrimer conjugate α-CDE and a man-made molecule L-HIPPO. (**a**) HIV-1 virion budding is mediated by viral protein Pr55^Gag^ that binds to a membrane phospholipid PIP2. PIP2-bound Pr55^Gag^ assembles in the membrane to form virions. (**b**) α-CDE delivers L-HIPPO into HIV-1-infected cell where L-HIPPO antagonizes the PIP2-Pr55^Gag^ binding to interfere with virus budding. The infecting HIV-1 is enclosed and dies with the apoptosis of the host cell. (**c**) Structures of PIP2, D-HIPPO, L-HIPPO, cyclodextrin/dendrimer conjugates (α-CDE), and α-CDE conjugated with TRITC (α-CDE-TRITC).

Generally, HIV recognizes and enters into CD4+ T lymphocytes. HIV proliferates inside the infected cells to release an enormous number of offspring virions that, in turn, propagates to other CD4+ T lymphocytes. Diminishing the release of new virions would help manage the virus population and reduce the burden of eradicating virus from the body. We sought to synthesize a small molecule that would hinder the budding of offspring virus, with the notion of turning an HIV-infected cell into a “prison cell” from which the invaded virus cannot escape (Fig. 1a and b). The host cell will eventually die without releasing the prisoner virus. Thus, if all reservoir viruses are kicked, locked in the host cell prison, and these cells apoptosed, the death of all such infected host cells would mark the eradication of HIV in the body.

It is known that the viral protein Pr55^Gag^ is one of the products produced by hijacked host cells and mediates HIV-1 virion budding. Ordinarily, Pr55^Gag^ migrates from the cytoplasm to the plasma membrane and binds to a specific inositol phospholipid, PIP2, in the membrane^6^. It was shown that the N-terminal MA (matrix antigen) domain of Pr55^Gag^ interacts with PIP2 by prior NMR study^7^ and recent follow-up reports revising the role of acyl group of PIP2^8, 9^. PIP2-bound Pr55^Gag^ assembles each other in the membrane to form virions (Fig. 1a)^6^. As Pr55^Gag^-PIP2 binding triggers the virion budding, we considered that a small molecule that binds firmly to Pr55^Gag^ could antagonize PIP2 in the Pr55^Gag^ binding. Previously, we established a highly-sensitive *in vitro* assay to determine the dissociation constant (*K*_d_) for the binding of Pr55^Gag^ to phosphoinositide derivatives using surface plasmon resonance (SPR) sensor analysis^10, 11^. We found that both negatively-charged inositol phosphates and the lipophilic acyl chains of phosphoinositide were essential for a strong interaction with Pr55^Gag^ ^10^. As well, fully-phosphorylated inositol IP6 (phytic acid, “PA”) bound to the MA domain approximately 10 times stronger than IP3, the inositol head of PIP2^11^. Accounting for each of these details, we had synthesized an artificial phosphoinositide, DL-HIPPO (DL-Heptanoylphosphatidyl Inositol Pentakisphosphate), as an isomeric mixture (a 1:1 mixture of D-HIPPO and L-HIPPO) (Fig. 1c). This compound was shown to have a MA-binding affinity 70-fold stronger than that of the less phosphorylated PIP2 derivative^11^. This activity was considered sufficient to antagonize PIP2 to suppress the membrane localization of Pr55^Gag^.

**Figure 2:**
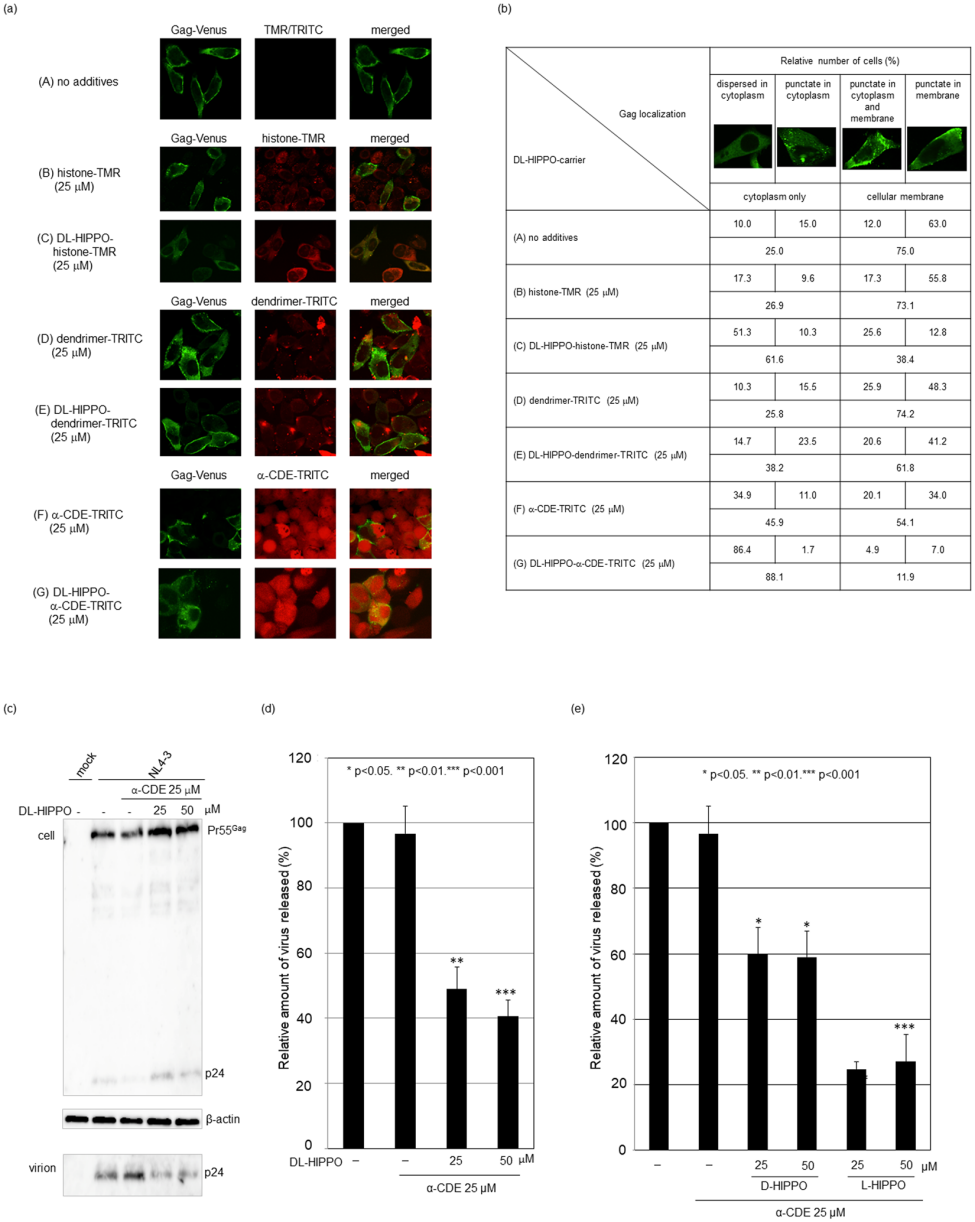
Effect of DL-HIPPO/D-HIPPO/L-HIPPO delivered into a cell by carrier on the cellular localization of Pr55^Gag^ and HIV-1 release. (**a**) Effect of DL-HIPPO delivered into a cell by various carriers on the cellular localization of Pr55^Gag^. (A) Cellular localization of intact Pr55^Gag^. HeLa cells were transfected with a plasmid vector to express Gag-Venus (pNL4-3/GagVenus) and, after 13 h, Gag localization was observed by fluorescence microscopy. (B, C) Effect of DL-HIPPO-histone on Gag localization. HeLa cells were transfected with pNL4-3/GagVenus and, after 10 h, a complex prepared from DL-HIPPO (25 μM) and histone conjugated with TMR (25 μM) (DL-HIPPO-histone-TMR) was added. After a further 3 h incubation, localization of Gag and histone was observed by fluorescence microscope. (D, E) Effect of DL-HIPPO-dendrimer on Gag localization. The same experiment as that of (B, C) was performed using dendrimer instead of histone. (F, G) Effect of DL-HIPPO-α-CDE on Gag localization. The same experiment as that of (B, C) was performed using α-CDE instead of histone. (**b**) Effect of DL-HIPPO-carrier on the cellular localization of Pr55^Gag^. A total of approximate 100 cells in each experiment of (**a**) were observed and categorized into 4 types “dispersed in cytoplasm”, “punctate in cytoplasm”, “punctate in cytoplasm and membrane”, and “punctate in membrane” shown at the top of the table. The relative number of cells (%) in each category is shown. (**c**) Effect of DL-HIPPO-α-CDE on HIV-1 release. HeLa cells were transfected with pNL4-3 and, after 10 h, complex prepared from DL-HIPPO (25 or 50 μM) and α-CDE (25 μM) (DL-HIPPO-α-CDE) was added. After a further 3 h incubation, supernatant and the cells were harvested, lysed, and analyzed by immunoblotting using anti-p24 antibody. Wider images are shown in Supplementary Information F. (**d**) Quantification of effect of DL-HIPPO-α-CDE on HIV-1 release. Intensity of the bands in (**c**) were quantitated using ImageJ, and the amount of released virus, (amount of p24 in supernatant)/(amounts of p24 and Pr55^Gag^ in cells), was calculated. Relative value to that of a control without additives is shown as percent. Data from three different experiments are shown as means ± standard deviations. P values were determined using Student’s t test. (**e**) Quantification of effect of D-HIPPO-α-CDE and L-HIPPO-α-CDE on HIV-1 release. The same experiment (3 h incubation after addition of HIPPO-α-CDE) as that in (**c**) and (**d**) was performed using enantiomerically pure D-HIPPO and L-HIPPO. Data from three different experiments are shown as means ± standard deviations. P values were determined using Student’s t test.

Here we examined the localization of HIV-1 Pr55^Gag^ in HeLa cells using Venus fluorescent labeled Pr55^Gag^ protein. HeLa cells were transfected with the plasmid vector pNL4-3/GagVenus^12^ having Venus yellow fluorescent protein at the C-terminus of Pr55^Gag^ instead of a region called Pol of HIV-1 infectious clone pNL4-3^13^, and incubated for 13 h. The cells were then fixed and observed by fluorescent confocal microscopy. In the absence of DL-HIPPO, Pr55^Gag^ localized to “cytosol only” in 25.0% of cells and to “cellular membrane” in 75.0% of cells (Fig. 2a(A) and b(A)), well in accordance with previous findings^12^.

As DL-HIPPO, with its dense negative phosphate charges, was membrane non-permeable (data not shown), we sought to add an appropriate moiety possessing high positive charge in order to improve delivery of the compound across the cell membrane. We considered histone and dendrimer, known as PIP2 carriers^14^, and α-cyclodextrin/polyamidoamine dendrimer (G3) conjugate (α-CDE) (Fig. 1c)^15–17^ as potential carriers for the negatively charged molecule.

A histone complex, DL-HIPPO-histone-TMR, was prepared by mixing DL-HIPPO (25 μM) and histone conjugated with the fluorescent molecule tetramethylrhodamine [TMR, purchased from Echelon Biosciences (Salt Lake City, UT, USA)] (25 μM). A dendrimer complex (DL-HIPPO-dendrimer-TRITC) was prepared from DL-HIPPO (25 μM) and dendrimer conjugated with the fluorescent molecule tetramethylrhodamine isothiocyanate (TRITC)^18^ (25 μM). These complexes were added to the transfected cells. The cells were incubated for 3 h, fixed, and observed by fluorescence microscopy. When DL-HIPPO-histone-TMR was added, the ratio of Pr55^Gag^ localized in “cytoplasm only” and “cellular membrane” were 61.6% and 38.4%, respectively, whereas Pr55^Gag^ localized mainly in “cellular membrane” (73.1%) in the control experiment using histone-TMR alone (Fig. 2a(B)(C) and b(B)(C)). On the other hand, DL-HIPPO-dendrimer-TRITC resulted in less efficient effect compared with the histone complex (Fig. 2a(D)(E) and b(D)(E)).

The third candidate carrier, α-CDE^17, 19^, induced the most favorable ratio of Pr55^Gag^ localization. α-CDE-TRITC was prepared from α-CDE according to a previous method^20^. α-CDE-TRITC and DL-HIPPO were mixed to form DL-HIPPO-α-CDE-TRITC, which was then added to HeLa cells. The ratio of Pr55^Gag^ localized in “cytoplasm only” was 88.1% and that in “cellular membrane” was 11.9%, whereas the ratio of Pr55^Gag^ localization in “cytoplasm only” and “cellular membrane” were 45.9% and 54.1%, respectively, in the experiment using α-CDE-TRITC alone (Fig. 2a(F)(G) and b(F)(G)). This weaker inhibitory effect would be traced back to that cyclodextrin abstracts cholesterol from cellular membrane and suppresses HIV-1 assembly^21^.

As DL-HIPPO-α-CDE-TRITC kept the most Pr55^Gag^ away from the membrane of the tested candidates, its capability to lock the trigger for virion budding was examined more extensively. HeLa cells were transfected with the HIV-1 infectious clone pNL4-3^13^ and incubated for 10 h. The DL-HIPPO-α-CDE-TRITC complex was added. After incubation for an additional 3 h, supernatant and cells were harvested and the amount of virus protein was analyzed by western blotting using anti-p24 antibody (Fig. 2c). This antibody reacts to both precursor protein Pr55^Gag^ and its cleavage product, p24. Nearly half of the HIV-1 release was suppressed for both concentrations (25 and 50 μM) of DL-HIPPO complex tested, although the amount of membrane localized Pr55^Gag^ was small (Fig. 2d).

Thus far we used DL-HIPPO that was synthesized as an equimolar mixture of D-HIPPO and L-HIPPO^11^. Usually, the biological activity of each isomer differs, i. e., in the case of D-HIPPO and L-HIPPO one isomer is potent and the other is less potent. Thus, the both isomers D-HIPPO and L-HIPPO were separately synthesized to identify the potent isomer (for the detail of synthesis, see Supplementary Information A).

Binding affinities of these two isomeric compounds to the MA domain of Pr55^Gag^ were determined using the same SPR assay used previously^10, 11^. The *K*_d_ for MA binding of D-HIPPO and L-HIPPO were 1.09 ± 0.47 and 0.18 ± 0.08 μM, respectively (Supplementary Information B). The *K*_d_ determined for DL-HIPPO (0.25 ± 0.17 μM)^11^ was between the two values. The non-natural L-compound showed a higher binding affinity to MA compared with the natural D-isomer. We further assessed the HIV-1 release suppressing activity of the each isomer. The complex prepared from L-HIPPO (25 or 50 μM) and α-CDE (25 μM), L-HIPPO-α-CDE, inhibited more than 70% of virus release, while the other isomer D-HIPPO-α-CDE suppressed just 40% of the release (Fig. 2e).

The non-natural L-isomer was shown to be the potent isomer both in Pr55^Gag^ binding and virus release suppression. To examine the possibility of apoptosis induction by L-HIPPO-α-CDE, we increased the incubation time beyond the three hours used in the virus-release experiment. Non-cytotoxicity of L-HIPPO-α-CDE to HeLa cells in the absence of HIV, even at high concentration (100 μM) and for prolonged incubation time (48 h), was shown by cell viability assay using MTT pigment. FACS analysis of cells treated with the complex using apoptosis indicator annexin V-Cy5 also confirmed the non-toxicity (for MTT assay and FACS, Supplementary Information C).

**Figure 3:**
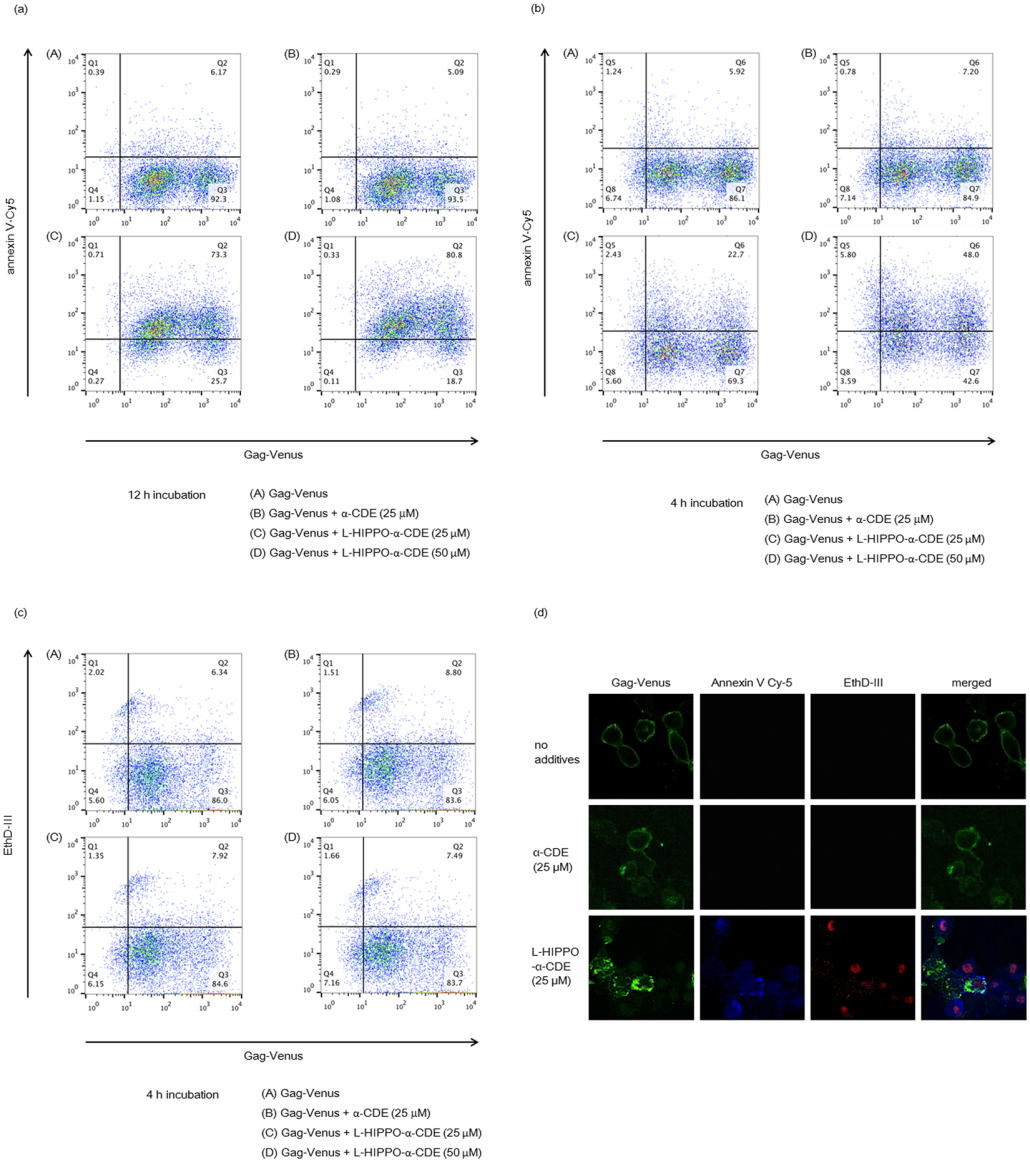
Apoptotic effect of L-HIPPO-α-CDE with HIV-1 protein. (**a**) Apoptotic effect. HeLa cells were transfected with pNL4-3/GagVenus, and after 10 h, complex prepared from L-HIPPO (25 or 50 μM) and α-CDE (25 μM) (L-HIPPO-α-CDE) was added. After a further 12 h incubation, FACS analysis using annexin V-Cy5 was performed. (**b**) Apoptotic effect after a shorter incubation time. The same experiment as that in (**a**) was performed using 4 h incubation instead of 12 h incubation. (**c**) Necrotic effect after a shorter incubation time. The same experiment as that in (**b**) was performed using EthD-III instead of annexin V-Cy5. (**d**) Apoptotic effect observed by microscopy. The same experiment as (**a**) using L-HIPPO (25 μM) and α-CDE (25 μM) was performed by microscopic observation using annexin V-Cy5 and EthD-III instead of FACS analysis.

**Figure 4:**
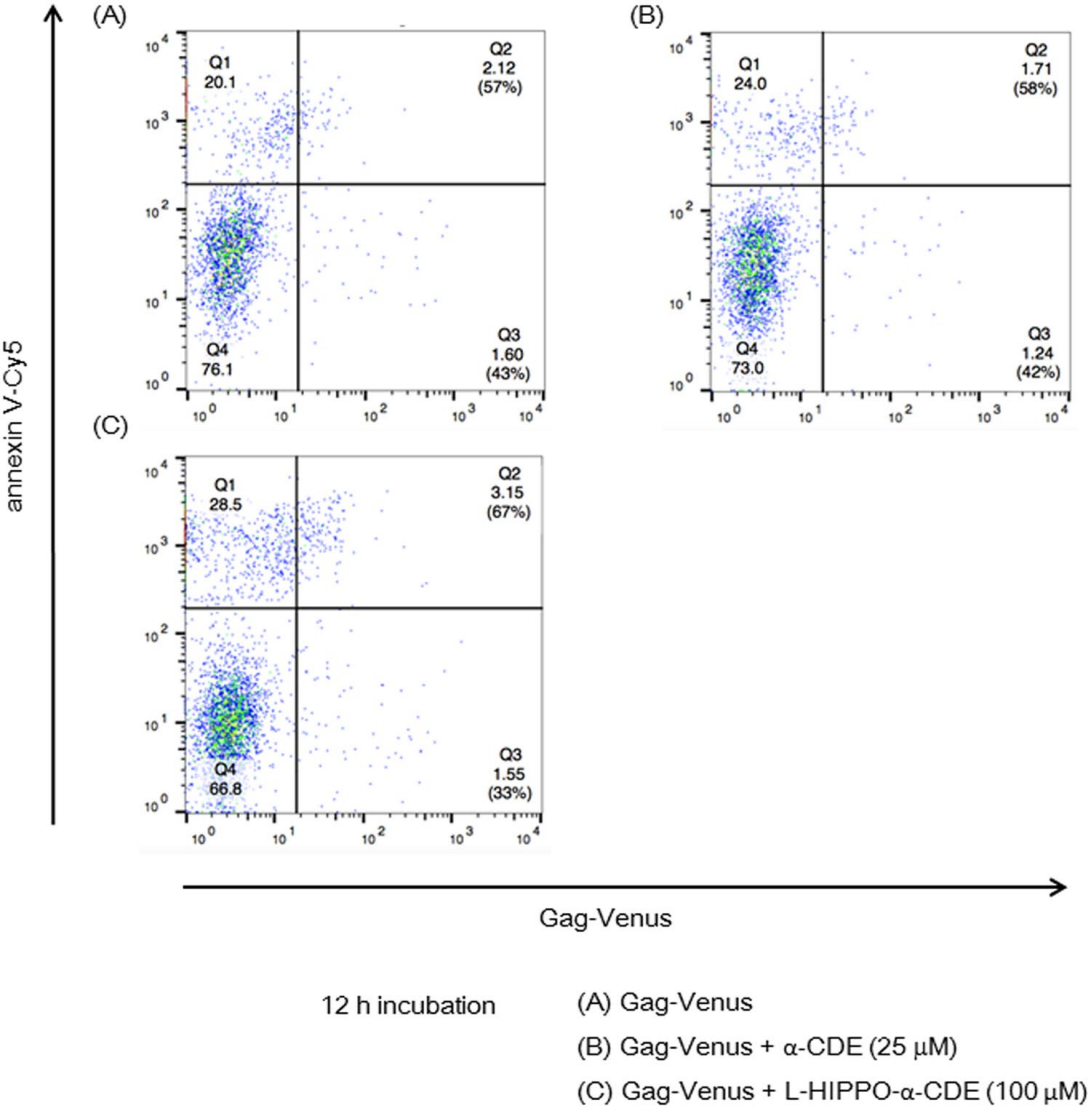
Apoptotic effect of L-HIPPO-α-CDE with HIV-1 protein in T cells. Jurkat cells were transfected with pNL4-3/GagVenus, and after 1 d, a complex prepared from L-HIPPO (100 μM) and α-CDE (25 μM) (L-HIPPO-α-CDE) was added. After a further 12 h incubation, FACS analysis using annexin V-Cy5 was performed.

The effect of L-HIPPO-α-CDE on HIV-1 protein was then examined. HeLa cells were transfected with pNL4-3/Gag Venus and incubated for 10 h. Cells were treated with L-HIPPO-α-CDE, incubated for a further 12 h, stained with annexin V-Cy5, and analyzed by FACS (Fig. 3a). The number of cells stained with annexin V was very low (Q2: 5–6%) in control experiments using cells transfected with the vector and untreated with additives or treated with only carrier α-CDE (25 μM). More than half of the transfected cells treated with L-HIPPO-α-CDE (25 or 50 μM) were stained with annexin V. A dose of 50 μM of the complex showed the highest ratio (Q2: 81%) of cells stained, indicating the induction of apoptosis. Considerable amounts of cells were stained with annexin V in experiments with a shorter incubation time (4 h) after addition of the complex (25 or 50 μM) (Fig. 3b). However, cell population staining with necrosis indicator EthD-III (ethidium homodimer III) was almost the same as that seen in the control (Fig. 3c), excluding necrosis and showing induction of apoptosis. Worthy of note, two populations, high population and accompanying dim population, were seen in the FACS analysis of the cells transfected with pNL4-3/Gag Venus. Decrease of transfection efficiency showed increment of dim population, demonstrating the dim population is extracellular Gag-Venus particles that bind to the untransfected cells (Supplementary Information D). Apoptosis of the dim population (Fig. 3a,b and Supplementary Information D) is thought to be due to bystander effect of apoptosis of Gag-Venus expressing cells. We also confirmed cell death by microscopic analysis, after addition of the complex (25 μM) and subsequent incubation of the transfected cells for 12 h. The cells were responsive to both annexin V and EthD-III, demonstrating late stage of apoptosis in the longer incubation (Fig. 3d). Similarly, we observed by FACS that 68% of the same cells were stained with EthD-III (data not shown). These results, taken together, show that L-HIPPO-α-CDE induced apoptosis of cells expressing HIV-1 protein. Finally, we examined whether this effect of L-HIPPO-α-CDE is observed in T cells by experiments using Jurkat cell line. As results, L-HIPPO-α-CDE (100 μM) did not show toxicity (Supplementary Information E), and in the presence of this complex, larger amount of cells (67%) were shown to induce apoptosis than those without L-HIPPO (58%) in a cell population expressing Gag-Venus (Fig. 4).

To sum up, we have developed a novel analogue of phosphoinositide L-HIPPO that binds to HIV-1 protein Pr55^Gag^ with strong affinity, and L-HIPPO-α-CDE complex suppresses membrane localization of Pr55^Gag^ and subsequent virus release and induces apoptosis of the host cell. Thus L-HIPPO-α-CDE encloses the penetrated virus components, locks them in the host cell, and destroy virus by means of apopmechantosis of the host cell. L-HIPPO is “non-natural” because of (i) L-type stereochemistry and (ii) IP6 structure with lipophilic group(s). Thus this “non-natural” compound would not conflict the intracellular function of natural inositol phospholipid such as PIP2. Actually, L-HIPPO did not show toxicity on cells in this study (Supplementary Information C). As impact of apoptosis on surrounding cells, caused by direct contact, release of soluble factors such as cytokines, and so on, has been widely studied and revealed well^22, 23^, its regulation would be possible. While bystander effect was observed in the apoptosis, this method would be improved so as to induce apoptosis without affecting other normal cells.

The “kick and kill” strategy begins with the use of LRA to activate (“kick”) the viral replication cycle^4^, and satisfiable “kill” device has not been available yet^4, 5^. In this study we developed an alternative method to terminate the fate of infected cells that is not “kill” but “suicide (apoptosis)”. For the future development of orally available L-HIPPO, a prodrug approach may be conceivable. The “lock-in and apoptosis” approach presented in this work suppresses the virus release and induces apoptosis to become bases of the eradication of HIV. Furthermore, this “lock-in and apoptosis” would pave the way for new anti-virus strategy against various viruses.

## Methods

### Plasmids, cells and transfection

A plasmid vector pNL4-3**/**GagVenus^12^ was used to express HIV-1 Gag conjugated with Venus yellow fluorescent protein at its C-terminus using pNL4-3^13^ as a full-length HIV-1 infectious clone. The human cervical cancer cell line HeLa and human leukemic T cell line Jurkat were maintained in Dulbecco’s modified Eagle’s medium supplemented with 5% heat-inactivated fetal bovine serum (FBS) and RPMI-1640 medium supplemented with 10% heat-inactivated FBS, respectively. Transfection of the HeLa cells with plasmids was performed using Lipofectamine 3000 (Life Technologies, Carlsbad, CA, USA), unless otherwise stated. Transfection of Jurkat cells with plasmids was performed by Amaxa Cell Line Nucleofector Kit V (Lonza, Basel, Switzerland).

### Synthesis and characterization of new compounds

See Supplementary Information.

### Preparation and addition of complexes of phosphoinositide analogues with carriers to cells

TMR Labeled Shuttle PIP Carrier 2 (Histone H1) (Histone-TMR) was purchased from Echelon Biosciences (Salt Lake City, UT, USA). Dendrimer-TRITC was prepared according to a previous method^18^. A conjugate of α-cyclodextrin and polyamidoamine dendrimer (G3) (α-CDE) was synthesized from monotosyl-α-cyclodextrin and dendrimer (G3) as previously described^17, 19^. α-CDE-TRITC was prepared from α-CDE according to a previous method^20^. Each carrier and DL-HIPPO, D-HIPPO, or L-HIPPO were mixed to form complexes, and added to HeLa cells. The cells were incubated at 37 °C for 15 min, washed with PBS (×1), and fresh medium was added.

### Fluorescence microscopy

Microscopic observations were performed using a Zeiss LSM 700 laser-scanning confocal microscopy (Carl Zeiss, Oberkochen, Germany), as previously described^24^.

### Immunoblot analysis

Supernatant and cell lysate, prepared by the PBS-Laemmli buffer method, were analyzed by immunoblotting as previously described^25^. HIV-1 p24 Gag monoclonal (#24-4) (NIH AIDS Research and References Reagent Program)^26, 27^ (1:1000) and anti-β-actin clone AC-15 (Sigma-Aldrich, St Louis, MO, USA) were used as a detection antibody. Immunoreactivity was detected by chemiluminescence using ImmunoStar LD (Wako Pure Chemical Industries, Osaka, Japan). Intensity of the bands were quantitated using ImageJ software.

### SPR studies

Dissociation constants (*K*d) were determined by competition assay using a BIACORE 2000 (GE Healthcare, Uppsala, Sweden). In this analysis, biotinylated D-*myo*-inositol-1,3,4,5-tetrakisphosphate^28^ was immobilized to a streptavidin conjugated sensor chip. A solution of MA protein, prepared from pEF-Gag (p17) cFLAG^10^ vector-transfected 293 T cells, was applied to the chip using flow buffer [10 mM HEPES, 150 mM NaCl, 3.4 mM EDTA, 0.005% Tween 20, 2% (v/v) glycerol, 0.5 mg mL^−1^ BSA, and 5% DMSO (pH 7.8)]. The details of this protocol have been described previously^11^.

### MTT assay

The MTT assay was performed as described previously^29^.

### FACS analysis

The cells were detached from the plate by 0.05% trypsin, washed with PBS, and then incubated with annexin V-Cy5 (BioVision, Milpitas, CA, USA) or ethidium homodimer III (Takara Bio, Kusatsu, Japan) for 10 min. After washing the cells with annexin-binding buffer (BioVision), the cells were fixed with 2% paraformaldehyde, washed with PBS again, and analyzed using a BD FACSCalibur (Becton Dickinson, NJ, USA).

## Electronic supplementary material

Supplementary Information
