# Supplementary material for: A clue to unprecedented strategy to HIV eradication: “Lock-in and apoptosis”
Source: Sci Rep. 2017 Aug 21;7:8957. doi: 10.1038/s41598-017-09129-w (PMC5567282; doi:10.1038/s41598-017-09129-w)

Supplementary Information

A clue to unprecedented strategy to HIV eradication: "Lock-in and apoptosis"

Hiroshi Tateishi[1], Kazuaki Monde[2], Kensaku Anraku[3], Ryoko Koga[1], Yuya Hayashi[4], Halil Ibrahim Ciftci[1], Hasan DeMirci[5,6], Taishi Higashi[4], Keiichi Motoyama[4], Hidetoshi Arima[4], Masami Otsuka[1*] & Mikako Fujita[7*]

[1]Department of Bioorganic Medicinal Chemistry, Faculty of Life Sciences, Kumamoto University, Kumamoto, Japan.

[2]Department of Microbiology, Faculty of Life Sciences, Kumamoto University, Kumamoto, Japan.

[3]Department of Medical Technology, Kumamoto Health Science University, Kumamoto, Japan.

[4]Department of Physical Pharmaceutics, Faculty of Life Sciences, Kumamoto University, Kumamoto, Japan.

[5]Stanford PULSE Institute, SLAC National Accelerator Laboratory, Menlo Park, California, USA.

[6]Biosciences Division, SLAC National Accelerator Laboratory, Menlo Park, California, USA.

[7]Research Institute for Drug Discovery, School of Pharmacy, Kumamoto University, Kumamoto, Japan.

Supplementary Information A:

Preparation of D-HIPPO [(-)-1] and L-HIPPO [(+)-2]

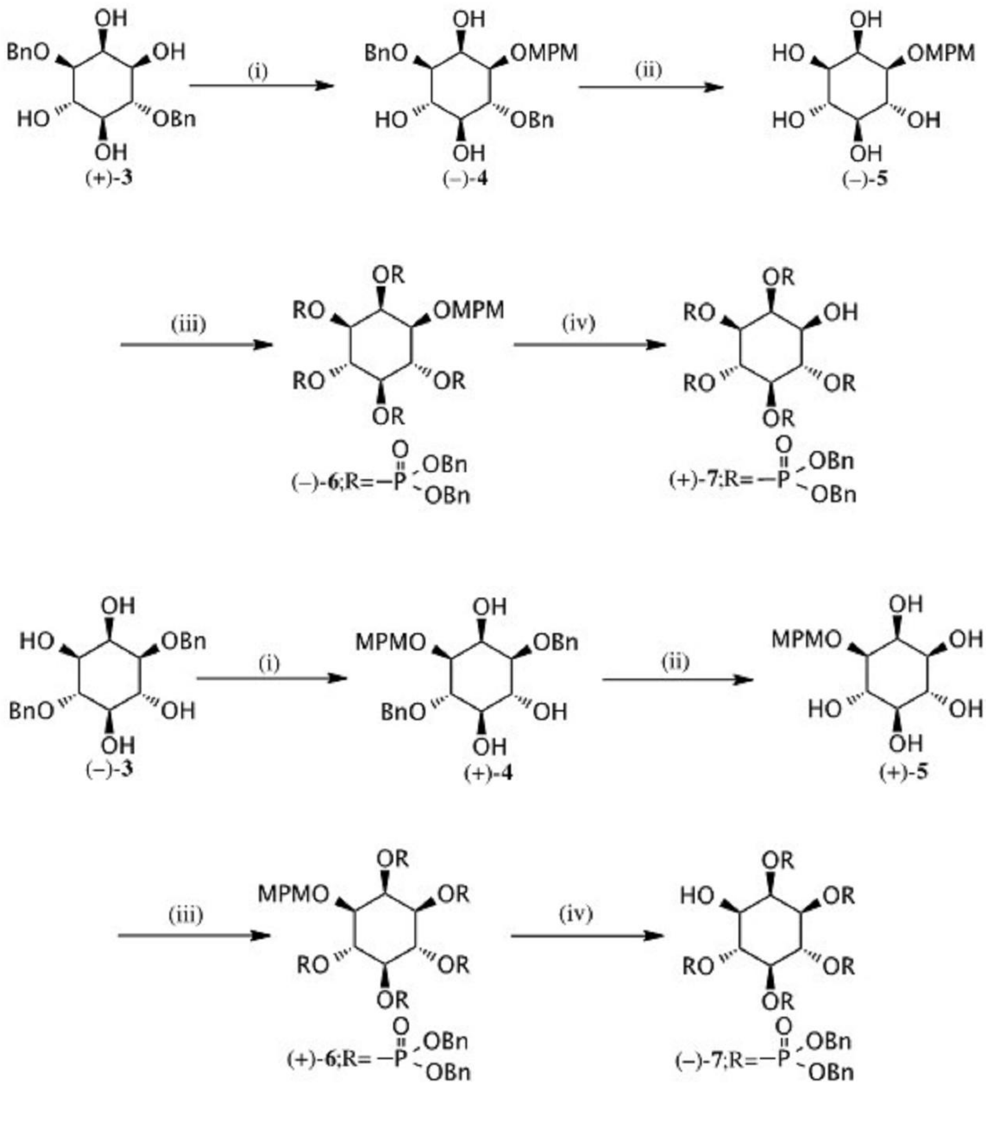

Reagents and conditions: (i) (a) Bu_2SnO, toluene, reflux, 3 h; (b) CsF, MPN-Cl, FMD, -78 ˚C then r.t., 48 h, (-)-**4** (85%), (+)-**4** (93%). (ii) H_2/W-2 Raney-Ni, MeOH, 50 ˚C, 1 h, (-)-**5** (75%), (+)-**5** (65%). (iii) (a) dibenzyl *N*,*N*-diethyl phosphoramidite, 1*H*-tetrazole, CH_2Cl_2, r.t., overnight; (b) *m*CPBA, CH_2Cl_2, -78 ˚C then r.t., 1 h, (-)-**6** (80%), (+)-**6** (80%). (iv) CAN, CH_3CN-H_2O, r.t., 1 h, (+)-**7** (64%), (-)-**7** (62%).

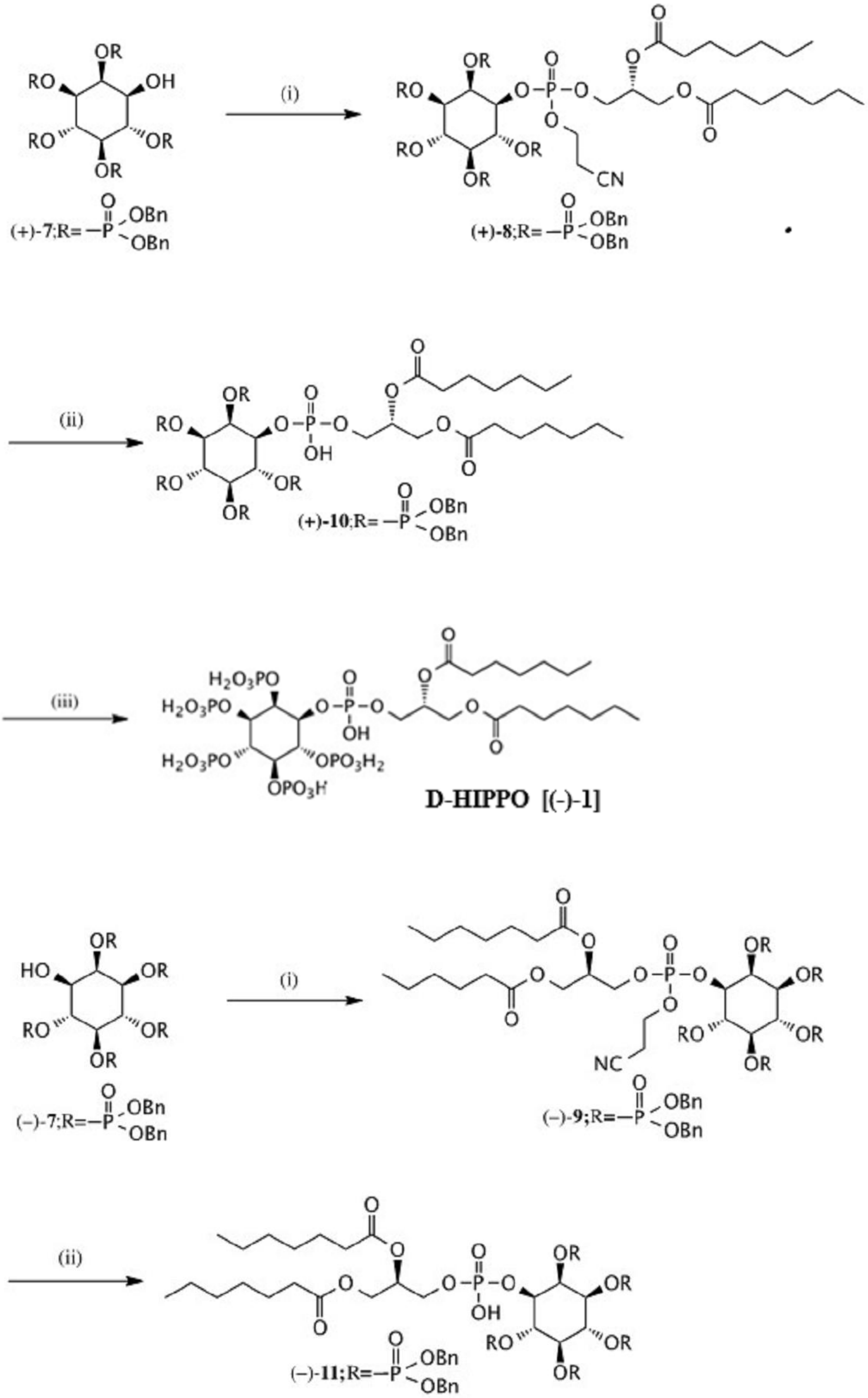

(i)
(+)-7;R=
(+)-8;R=
(ii)
(+)-10;R=
(iii)
D-HIPPO [(-)-1]
(i)
(–)-7;R=
(–)-9;R=
(ii)
(–)-11;R=

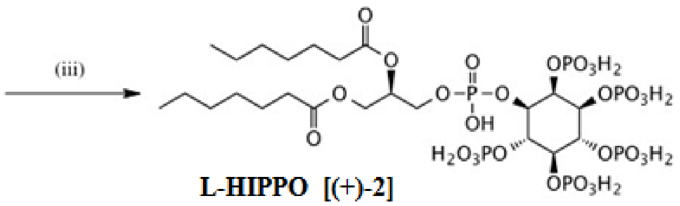

Reagents and conditions: (i) (a) 1,2-Di-*O*-heptanoyl-glycerol, (2-cyanoethyl)-*N*, *N*, *N'*, *N'*-tetraisopropylphosphoramidite, 1*H*-tetrazole, CH2Cl_2, r.t., 1 h; (b) (+)-**7** or (-)-**7**, 1*H*-tetrazole, CH_2Cl_2, r.t., overnight; (c) *tert*-BuOOH, CH_2Cl_2, r.t., 5 min, (+)-**8** (69%), (-)-**9** (62%). (ii) Et_3N, CH_2Cl_2, r.t., overnight, (+)-**10** (71%), (-)-**11** (88%). (iii) H2/Pd-C, *t*-BuOH-H_2O, r.t., 24 h, D-HIPPO [(-)-**1**] (40%), L-HIPPO [(+)-**2**] (43%).

General methods

Chemicals were purchased from Aldrich, Fluka, Kanto Chemical, Nacalai tesque, and Wako. Thin layer chromatography (TLC) was performed on precoated plates (Merck TLC sheets silica 60 F_{254}): products were visualized by spraying phosphomolybdic acid in EtOH or basic potassium permanganate and heated at high temperature. Chromatography was carried out on Silica Gel 60 N (40–100 mesh). Reverse phase chromatography was performed using a C_{18} column (Cole-Parmer, USA). Cation exchange chromatography was performed using Dowex 50WX8 (H^+, 100-200 mesh). NMR spectra (JEOL JNM-AL300 or BRUKER AVANCE 600) were referenced to $SiMe_4$ or HDO. Infrared spectra were recorded on a JASCO FT/IR-410. The samples were prepared as KBr discs or thin films between sodium chloride discs. Micro-analysis was carried out using a Yanaco MT-3S. High resolution MS (HRMS) were recorded with a JEOL JMS-DX303HF by using positive and negative FAB with 3-nitrobenzyl alcohol (NBA) (containing HMPA or not) as the matrix. Optical rotation values were measured in a 5 cm cell with JASCO DIP-1000.

D-3,6-Di-*O*-benzyl-1-*O*-(*p*-methoxybenzyl)-*myo*-inositol (–)-4

A mixture of **(+)-3**[1] (0.88 g, 2.44 mmol) and dibutyltin oxide (0.79 g, 3.14 mmol) in toluene (30 ml) was refluxed for 3h in a Dean-Stark apparatus to remove water [2]. The mixture was concentrated under reduced pressure. To the residue was added cesium fluoride (0.74 g, 4.88 mmol), and the mixture was suspended in heated DMF (30 ml) at 100 °C. To the resulting solution was added *p*-methoxybenzyl chloride (0.40 ml, 2.93 mmol) at –78 °C, and the mixture was stirred at room temperature under argon for 48 h [3]. After concentration of the reaction mixture under reduced pressure, the residue was purified

by silica gel column chromatography (CH_2Cl_2–MeOH=10:1) to afford **(–)-4** (1.00 g, 85%) as a white solid.

^{1}H NMR ($CDCl_3$) δ: 2.47 (1H, bs, OH), 2.62 (2H, bs, OH), 3.22 (1H, dd, *J* = 9.3, 2.7 Hz, CH), 3.37 (1H, dd, *J* = 9.3, 2.7 Hz, CH), 3.39 (1H, t, *J* = 9.3 Hz, CH), 3.78–3.82 (4H, m, OCH_3, CH), 3.96 (1H, t, *J* = 9.3 Hz, CH), 4.21 (1H, t, *J* = 2.7 Hz, CH), 4.61–4.97 (6H, m, CH_2 × 3), 6.86 (2H, d, *J* = 8.5 Hz, $CH_3OC_6\underline{H_4}$(CH x 2)), 7.23–7.42 (12H, m, C_6H_5 × 2, $CH_3OC_6\underline{H_4}$(CH × 2)).

^{13}C NMR ($CDCl_3$) δ: 55.3, 66.9, 71.9, 72.1, 72.2, 74.1, 75.5, 79.0, 79.4, 80.4, 113.9, 127.8, 127.9, 128.0, 128.5, 128.6, 129.5, 129.8, 137.7, 138.7, 159.4, 164.3.

IR (KBr) 3600, 3100, 1610, 1520, 1450, 1050, 810, 750 cm^{-1}.

HRMS(FAB) *m/z* calcd for $C_{28}H_{32}O_7Na$ $(M+Na)^+$ 503.2046 Found:503.2078.

Anal. Calcd for $C_{28}H_{32}O_7$: C, 69.98; H, 6.71. Found: C, 69.86; H, 6.58.

TLC; R_f 0.50 (CH_2Cl_2–MeOH=10:1).

$[\alpha]_D^{20}$= –8.4 (c=1 in MeOH)

L-1,4-Di-*O*-benzyl-3-*O*-(*p*-methoxybenzyl)-*myo*-inositol (+)-4

(–)-3[1] (0.42 g, 1.18 mmol) was allowed to react, under the same conditions as described for the preparation of **(–)-4**, to give **(+)-4** (0.52 g, 93%) as a white solid.

^{1}H NMR ($CDCl_3$) δ: 2.78 (1H, bs, OH), 3.15 (1H, dd, *J* = 9.5, 2.6 Hz, CH), 3.27-3.42 (4H, m, CH x 2, OH x 2), 3.74-3.78 (4H, m, CH_3, CH), 3.93 (1H, t, *J* = 9.5 Hz, CH), 4.13 (1H, t, *J* = 2.4 Hz, CH), 4.56–4.90 (6H, m, CH_2 × 3), 6.82 (2H, d, *J* = 8.6 Hz, $CH_3OC_6\underline{H_4}$(CH x 2)), 7.20–7.34 (12H, m, C_6H_5 × 2, $CH_3OC_6\underline{H_4}$(CH × 2)).

^{13}C NMR ($CDCl_3$) δ: 55.1, 66.9, 71.8, 71.9, 72.1, 74.2, 75.2, 78.8, 79.2, 80.4, 113.8, 127.4, 127.8, 128.2, 128.4, 129.4, 129.8, 137.7, 138.7, 159.2.

IR (KBr) 3600, 3100, 2870, 1610, 1520, 1450, 1050, 810, 750 cm^{-1}.

HRMS(FAB) *m/z* calcd for $C_{28}H_{32}O_7Na$ $(M+Na)^+$ 503.2046 Found:503.2097.

Anal. Calcd for $C_{28}H_{32}O_7$: C, 69.98; H, 6.71. Found: C, 69.74; H, 6.63.

TLC; R_f 0.50 (CH_2Cl_2–MeOH=10:1).

$[\alpha]_D^{20}$= 7.0 (c=1 in MeOH)

D-1-*O*-(*p*-Methoxybenzyl)-*myo*-inositol (–)-5

To a solution of **(–)-4** (0.97 g, 2.02 mmol) in MeOH (10 ml) was added W-2 RANEY® Nickel (0.20 g, 3.03 mmol), and the resulting mixture was stirred at 50 °C under hydrogen for 1 h [4]. The mixture was filtrated through pad of celite and concentrated under reduced pressure. The residue was washed

with heated AcOEt, and the resulting crystals were filtrated. Drying of the crystals under reduced pressure afforded **(–)-5** (0.46 g, 75%) as a white solid.

^{1}H NMR (DMSO) δ: 2.93 (1H, t, J = 9.2 Hz, CH), 3.06 (2H, dd x 2, J = 9.7, 2.3 Hz, CH x 2), 3.35 (1H, t, J = 9.3 Hz, CH), 3.45 (1H, t, J = 9.3 Hz, CH), 3.69 (3H, s, OCH_3), 3.83-3.85 (1H, m, CH), 4.40–4.53 (2H, m, CH_2), 6.84 (2H, d, J = 8.2 Hz, $CH_3OC_6\underline{H_4}$(CH × 2)), 7.27 (2H, d, J = 8.2 Hz, $CH_3OC_6\underline{H_4}$(CH × 2)).

^{13}C NMR (DMSO) δ: 55.0, 69.3, 70.3, 71.8, 72.0, 72.5, 75.4, 79.6, 113.4, 129.1, 131.2, 158.5.

IR (KBr) 3390, 2910, 1610, 1590, 1510, 1250, 1120, 890, 820 cm^{-1}.

HRMS(FAB) *m/z* calcd for $C_{14}H_{20}O_7Na$ $(M+Na)^+$ 323.1107 Found:323.1109.

Anal. Calcd for $C_{14}H_{20}O_7$: C, 55.99; H, 6.71. Found: C, 56.03; H, 6.85.

TLC; R_f 0.39 (CH_2Cl_2–MeOH = 3 : 1).

$[\alpha]_D^{20}$= –11.7 (c=1 in DMSO)

L-3-*O*-(*p*-Methoxybenzyl)-*myo*-inositol (+)-5

(+)-4 (0.53 g, 1.09 mmol) was allowed to react, under the same conditions as described for the preparation of **(–)-5**, to give **(+)-5** (0.21 g, 65%) as a white solid.

^{1}H NMR (DMSO) δ: 2.94 (1H, t, J = 9.2 Hz, CH), 3.06 (2H, dd x 2, J = 9.0, 2.2 Hz, CH x 2), 3.35 (1H, t, J = 9.3 Hz, CH), 3.45 (1H, t, J = 9.3 Hz, CH), 3.68 (3H, s, OCH_3), 3.90-3.95 (1H, m, CH), 4.39–4.52 (7H, m, OH × 5, CH_2), 6.84 (2H, d, J= 8.4 Hz, $CH_3OC_6\underline{H_4}$(CH × 2)), 7.27 (2H, d, J= 8.4 Hz, $CH_3OC_6\underline{H_4}$(CH × 2)).

^{13}C NMR (DMSO) δ: 55.0, 69.3, 70.3, 71.8, 71.9, 72.0, 72.5, 72.8, 75.4, 79.6, 113.4, 129.1, 131.2, 158.5.

IR (KBr) 3700, 2800, 1610, 1590, 1510, 1250, 1170, 890, 820 cm^{-1}.

HRMS(FAB) *m/z* calcd for $C_{14}H_{20}O_7Na$ $(M+Na)^+$ 323.1107 Found:323.1107.

TLC; R_f 0.39 (CH_2Cl_2–MeOH = 3 : 1).

$[\alpha]_D^{20}$= 11.4 (c=1 in DMSO)

D-1-*O*-(*p*-Methoxybenzyl)-2,3,4,5,6-penta-*O*-dibenzylphosphoryl-*myo*-inositol (–)-6

To suspension of **(–)-5** (0.1 g, 0.33 mmol) in CH_2Cl_2 (10 ml) was added MS4A, and the resulting suspension was stirred at room temperature under argon for 15 min. To the mixture was added dibenzyl *N*,*N*-diethyl phosphoramidite (1g, 3.32 mmol) folowed by 1*H*-terazole (0.23 g, 3.32 mmol), the resulting mixture was stirred at room temperature under argon for overnight. To the mixture was added *m*-chloroperbenzoic acid (0.57 g, 3.30 mmol) in small portions, and the resulting mixture was stirred

–78 °C to room temperature for 1 h. The mixture was purified by silica gel column chromatography (Hexane-AcOEt=1:2) to afford **(–)-6** (0.43 g, 80%) as a colorless oil.

^{1}H NMR ($CDCl_3$) δ: 3.56 (1H, d, *J* = 9.9Hz, CH), 3.66 (3H, s, CH_3), 4.41 (1H, t, J=9.5, CH), 4.43 (1H, d, *J* = 10.3Hz, CH), 4.57 (1H, dd, *J* = 19.4, 9.5 Hz, CH), 4.66 (1H, d, *J* = 10.6Hz, CH), 4.74-5.24 (22H, m, $C\underline{H_2}C_6H_5$ x 10, CH x 2), 5.54 (1H, d, *J* = 9.5Hz, CH), 6.68 (2H, d, *J* = 8.8, $C_6\underline{H_4}$(CH x 2)), 7.06-7.35 (52H, m, C_6H_5 x 10, $C_6\underline{H_4}$(CH x 2)).

^{13}C NMR ($CDCl_3$) δ: 55.0, 69.1, 69.2, 69.4, 69.5, 69.6, 69.7, 69.8, 69.9, 71.7, 73.4, 73.8, 74.9, 76.3, 76.6, 113.5, 127.6, 127.7, 127.8, 127.8, 128.0, 128.1, 128.2, 128.3, 128.4, 129.4, 129.8, 130.5, 132.5, 135.5, 135.6, 135.7, 135.8, 135.9, 136.0, 166.6.

IR (neat) 2970, 1710, 1610, 1515, 1500, 1280, 1215, 1014, 880, 740, 700 cm^{-1}.

HRMS(FAB) *m/z* calcd for $C_{84}H_{85}O_{22}P_5Na$ $(M+Na)^+$ 1623.4118 Found:1623.4196.

TLC; Rf 0.40 (Hexane-AcOEt=1:2).

$[\alpha]_D^{20}$= –0.8 (c=1 in $CHCl_3$)

L-3-*O*-(*p*-Methoxybenzyl)-1,2,4,5,6-penta-*O*-dibenzylphosphoryl-*myo*-inositol (+)-6

(+)-5 (0.1 g, 0.33 mmol) was allowed to react, under the same conditions as described for the preparation of **(–)-6**, to give **(+)-6** (0.42 g, 80%) as a colorless oil.

^{1}H NMR ($CDCl_3$) δ: 3.55 (1H, d, *J* = 9.9 Hz, CH), 3.67 (3H, s, CH_3), 4.37 (1H, d, *J* = 9.7 Hz, CH), 4.42 (1H, *J* = 10.4 Hz, CH), 4.57 (1H, dd, *J* = 19.4, 9.7 Hz, CH), 4.65 (1H, d, *J* = 9.7 Hz, CH), 4.36-5.23 (20H, m, $C\underline{H_2}C_6H_5$ x 10), 5.52 (1H, d, *J* = 9.3 Hz, CH), 6.67 (1H, d, *J* = 8.4, $C_6\underline{H_4}$(CH x 2)), 7.07-7.35 (52H, m, C_6H_5 x 10, $C_6\underline{H_4}$(CH x 2)).

^{13}C NMR ($CDCl_3$) δ: 55.0, 69.1, 69.2, 69.4, 69.6, 69.7, 69.8, 69.9, 71.8, 73.5, 73.9, 75.0, 76.3, 76.5, 113.6, 127.6, 127.7, 127.9, 128.0, 128.1, 128.2, 128.3, 128.4, 128.5, 129.4, 129.8, 130.5, 132.5, 135.5, 135.6, 135.7, 135.8, 135.9, 136.0, 159.3.

IR (neat) 3030, 2970, 2900, 2835, 1710, 1610, 1515, 1500, 1280, 1215, 1015, 880, 740, 700 cm^{-1}.

HRMS(FAB) *m/z* calcd for $C_{84}H_{85}O_{22}P_5Na$ $(M+Na)^+$ 1623.4118 Found:1623.4087.

Anal. Calcd for $C_{84}H_{85}O_{22}P_5$: C, 63.00; H, 5.35. Found: C, 63.12; H, 5.53.

TLC; Rf 0.40 (Hexane-AcOEt=1:2).

$[\alpha]_D^{20}$= 0.6 (c=1 in $CHCl_3$)

D-2,3,4,5,6-Penta-*O*-dibenzylphosphoryl-*myo*-inositol (+)-7

To a solution of **(-)-6** (0.16g, 0.10 mmol) in CH_3CN-H_2O (9:1, 5 ml) was added diammonium cerium(IV) nitrate (0.12 g, 0.21 mmol)[5] and the resulting mixture was stirred at room temperature for 1 h. The resulting mixture was concentrated under reduced pressure, and the residue was purified by

silica gel column chromatography (CH_2Cl_2-AcOEt=1:5) to afford **(+)-7** (0.10 g, 64%) as a colorless oil.

^{1}H NMR ($CDCl_3$) δ: 4.03 (1H, d, *J* = 8.4 Hz, CH), 4.57 (1H, t, *J* = 9.2 Hz, CH), 4.61-5.29 (23H, m, C$\underline{H_2}$$C_6H_5$ x 10, CH x 3), 5.40 (1H, d, *J* = 8.8 Hz, CH), 5.65 (1H, bs, OH), 7.07-7.35 (50H, m, C_6H_5 x 10).

^{13}C NMR ($CDCl_3$) δ: 69.0, 69.4, 69.5, 69.6, 69.7, 69.8, 70.0, 70.1, 73.6, 74.9, 76.5, 77.7, 78.9, 127.5, 127.8, 127.9, 128.0, 128.1, 128.2, 128.3, 128.4, 129.4, 129.9, 132.6, 134.2, 135.4, 135.6, 135.7, 135.8, 136.0, 167.0.

IR (neat) 3255, 3030, 2925, 1610, 1500, 1455, 1280, 1215, 1015, 880, 740, 700 cm^{-1}.

HRMS(FAB) *m/z* calcd for $C_{84}H_{85}O_{22}P_5Na$ $(M+Na)^+$ 1503.3543 Found:1503.3604.

TLC; Rf 0.31 (CH_2Cl_2-AcOEt=1:5).

$[\alpha]_D^{20}$= 14.5 (c=1 in $CHCl_3$)

L-1,2,4,5,6-Penta-*O*-dibenzylphosphoryl-*myo*-inositol (–)-7

(+)-6 (0.30 g, 0.19 mmol) was allowed to react, under the same conditions as described for the preparation of **(+)-7**, to give **(-)-7** (0.17 g, 62%) as a colorless oil.

^{1}H NMR ($CDCl_3$) δ: 3.86 (1H, d, *J* = 8.1 Hz, CH), 4.39-4.68 (3H, m, CH x 3), 4.84-5.26 (21H, m, C$\underline{H_2}$$C_6H_5$ x 10, CH), 5.34 (1H, d, *J* = 8.4 Hz, CH), 5.51 (1H, bs, OH), 7.10-7.34 (50H, m, C_6H_5 x 10).

^{13}C NMR ($CDCl_3$) δ: 69.3, 69.4, 69.5, 69.6, 69.8, 69.9, 70.0, 70.1, 70.2, 73.7, 76.6, 77.4, 79.1, 127.8, 127.9, 128.1, 128.2, 128.3, 128.4, 128.5, 128.6, 129.9, 135.5, 135.6, 135.8, 135.9, 136.9, 160.5.

IR (neat) 3320, 3065, 3035, 2960, 1720, 1500, 1455, 1280, 1215, 1015, 880, 740, 700 cm^{-1}.

HRMS(FAB) *m/z* calcd for $C_{84}H_{85}O_{22}P_5Na$ $(M+Na)^+$ 1503.3543 Found:1503.3535.

TLC; Rf 0.31 (CH_2Cl_2-AcOEt=1:5).

$[\alpha]_D^{20}$= –13.9 (c=1 in $CHCl_3$)

D-1-*O*-[(1’,2’-Di-*O*-heptanoyl-*sn*-glycero)cyanoethyl]phosphoryl-2,3,4,5,6- pentakis-*O*-dibenzylphosphoryl-*myo*-inositol (+)-8

The mixture of 1,2-Di-O-heptanoyl-sn-glycerol (0.051 g, 0.16 mmol) in CH_2Cl_2 (5 ml) was added (2-cyanoethyl)-*N,N,N’,N’*-tetraisopropylphosphoramidite (0.051 ml, 0.16 mmol) followed by MS4A, and the resulting mixture was stirred at room temperature under argon for 15 min. To the mixture was added 1*H*-tetrazole (0.011 g, (0.16 mmol), and the resulting mixture was stirred at room temperature under argon for 10 min. To the mixture was added, completely dissolved compound **(+)-7** (0.052 g, 0.032 mmol) in CH_2Cl_2 (5 ml) with MS4A, followed by adding 1*H*-tetrazole (0.011 g, 0.16 mmol), and the resulting mixture was stirred at room temperature for 24 h. To the mixture was added *tert*-

butylhydroperoxide (0.032 ml, 0.32 mmol), and stirred at room temperature for further 5 min. The mixture was purified by silica gel column chromatography (hexane-acetone=2:1) to afford **(+)-8** (0.042 g, 69%) as a yellow oil.

^{1}H NMR ($CDCl_3$) δ: 0.86 (6H, t, *J* = 6.6 Hz, CH_3 x 2), 1.14-1.38 (12H, m, CH_2 x 6), 1.55 (4H, quin, *J* = 6.6 Hz, CH_2 x 2), 2.17-2.26 (4H, m, CH_2), 2.51 (2H, t, *J* = 6.6 Hz, CNC$\underline{H_2}$), 4.13-4.54 (10H, m, C$\underline{H_2}$CHC$\underline{H_2}$, CH x 4, $CNCH_2$C$\underline{H_2}$), 4.89-5.28 (22H, m, C$\underline{H_2}C_6H_5$ x 10, CH x 2), 5.52-5.58 (1H, m, CH), 7.09-7.40 (50H, m, C_6H_5 x 10).

^{13}C NMR ($CDCl_3$) δ: 13.9, 22.4, 24.6, 24.7, 28.7, 31.3, 33.8, 33.9, 34.0, 61.5, 69.3, 69.6, 69.7, 69.8, 69.9,73.0, 73.7,74.5,75.1, 127.8, 127.9, 128.0, 128.1, 128.2, 128.3, 128.4, 128.5, 128.6, 128.7, 135.6, 135.7, 135.8, 135.9, 172.9, 173.2.

IR (neat) 3035, 2960, 1740, 1455, 1380, 1280, 1020, 880, 740, 700 cm^{-1}.

HRMS(FAB) *m/z* calcd for $C_{96}H_{111}O_{28}NP_6Na$ $(M+Na)^+$ 1934.5616 Found:1934.5614.

TLC; Rf 0.38 (hexane-acetone=2:1).

$[\alpha]_D^{20}$= 3.2 (c=1 in $CHCl_3$)

L-3-*O*-[(1’,2’-Di-*O*-heptanoyl-*sn*-glycero)cyanoethyl]phosphoryl-1,2,4,5,6- pentakis-*O*-dibenzylphosphoryl-*myo*-inositol (–)-9

(–)-7 (0.15 g, 0.099 mmol) was allowed to react, under the same conditions as described for the preparation of **(+)-8**, to give **(–)-9** (0.12 g, 62%) as a yellow oil.

^{1}H NMR ($CDCl_3$) δ: 0.86 (6H, t, *J* = 6.8 Hz, CH_3 x 2), 1.19-1.29 (12H, m, CH_2 x 6), 1.49-1.63 (4H, m, CH_2 x 2), 2.15-2.37 (4H, m, CH_2), 2.45-2.57 (2H, m, CNC$\underline{H_2}$), 4.00-4.36 (7H, m, C$\underline{H_2}CHCH_2$, CH x 3, $CNCH_2$C$\underline{H_2}$), 4.46-4.60 (3H, m, CH, $CNCH_2$C$\underline{H_2}$), 4.89-5.29 (22H, m, C$\underline{H_2}C_6H_5$ x 10, CH x 2), 5.53-5.55 (1H, m, CH), 7.10-7.39 (50H, m, C_6H_5 x 10).

^{13}C NMR ($CDCl_3$) δ: 14.0, 22.4, 24.8, 28.7, 31.4, 33.9, 34.0, 34.2, 61.4, 61.5, 69.7, 69.8, 73.1, 73.7, 73.9, 74.5,75.0, 75.3, 127.9, 128.0, 128.1, 128.2, 128.3, 128.4, 128.5, 135.6, 135.8, 135.9, 172.9, 173.2.

IR (neat) 2860, 1740, 1455, 1280, 1015, 880, 740, 700 cm^{-1}.

HRMS(FAB) *m/z* calcd for $C_{96}H_{111}O_{28}NP_6Na$ $(M+Na)^+$ 1934.5616 Found:1934.5569.

TLC; Rf 0.38 (hexane-acetone=2:1).

$[\alpha]_D^{20}$= –1.0 (c=1 in $CHCl_3$)

D-1-*O*-(1’,2’-Di-*O*-heptanoyl-*sn*-glycero)phosphatidyl-2,3,4,5,6-pentakis-*O*-dibenzylphosphoryl-*myo*-inositol (+)-10

To the solution of **(+)-8** (0.042 g, 0.022 mmol) in CH_3CN (3 ml) was added Et_3N (1 ml) and stirred for overnight. After concentration of the reaction mixture under reduced pressure, the residue was

purified by silica gel column chromatography (CH_2Cl_2-MeOH=9:1) to afford **(+)-10** (0.029 g, 71%) as a colorless oil.

^{1}H NMR ($CDCl_3$) δ: 0.83 (6H, t, J = 6.6 Hz, CH_3 x 2), 1.20-1.26 (12H, m, CH_2 x 6), 1.47-1.51 (4H, m, CH_2 x 2), 2.16 (2H, t, J = 7.7 Hz, CH_2), 2.23 (2H, t, J = 7.7 Hz, CH_2), 4.10-4.49 (9H, m, CH_2, CH x5), 4.54-5.30 (20H, m, $C\underline{H_2}C_6H_5$ x 10, CH), 5.47-5.53 (1H, m, CH), 6.10 (1H, d, J = 9.2 Hz CH), 6.85-7.35 (50H, m, C_6H_5 x 10).

^{13}C NMR ($CDCl_3$) δ: 14.0, 22.4, 24.7, 24.8, 28.7, 31.4, 34.0, 34.2, 62.6, 69.3, 70.0, 70.2, 70.3, 70.5, 70.6, 71.3, 73.4, 75.1, 75.3, 76.3, 127.2, 127.5, 127.8, 127.9, 128.0, 128.1, 128.2, 128.3, 128.4, 128.5, 128.8, 135.0, 135.1,135.9, 136.0, 136.1, 173.0, 173.2.

IR (neat) 3035, 2930, 1740, 1455, 1380, 1280, 1020, 880, 740, 690 cm^{-1}.

MS (FAB) *m/z* 1880.79 $(M + Na)^+$.

TLC; Rf 0.36 (CH_2Cl_2-MeOH=9:1).

$[\alpha]_D^{20}$= 5.6 (c=1 in $CHCl_3$)

L-3-*O*-(1',2'-Di-*O*-heptanoyl-*sn*-glycero)phosphatidyl-1,2,4,5,6-pentakis-*O*-dibenzylphosphoryl-*myo*-inositol (–)-11

(–)-9 (0.12 g, 1.04 mmol) was allowed to react, under the same conditions as described for the preparation of **(+)-10**, to give **(–)-11** (0.093 g, 88%) as a colorless oil.

^{1}H NMR ($CDCl_3$) δ: 0.86 (6H, t, J = 6.6 Hz, CH_3 x 2), 1.23-1.32 (12H, m, CH_2 x 6), 1.34-1.57 (4H, m, CH_2 x 2), 2.18 (2H, t, J = 7.7 Hz, CH_2), 2.26 (2H, t, J = 7.7 Hz, CH_2), 4.12-4.34 (4H, m, $CH_2CHC\underline{H_2}$, CH x 2), 4.62-4.68 (1H, dd, J = 11.4, 7.7 Hz, CH), 4.77-5.55 (23H, m, $C\underline{H_2}C_6H_5$ x 10, $C\underline{H_2}C\underline{H}CH_2$), 6.12 (1H, d, J = 9.2 Hz CH), 6.86-7.41 (50H, m, C_6H_5 x 10).

^{13}C NMR ($CDCl_3$) δ: 14.0, 22.4, 24.7, 24.8, 28.7, 31.4, 33.9, 34.2, 62.5, 64.0, 69.2, 69.3, 69.5, 69.7, 70.0, 70.2, 70.3, 70.4, 70.5, 70.6, 71.0, 71.1, 73.4, 75.2, 76.3, 127.6, 127.8, 127.9, 128.0, 128.1, 128.2, 128.3, 128.4, 128.5, 128.6, 128.7, 129.3, 135.0, 135.2, 135.4, 135.7, 135.8, 135.9, 136.0, 136.1, 136.3, 173.0, 173.2.

IR (neat) 3000, 2820, 1735, 1455, 1380, 1040, 740, 700 cm^{-1}.

MS (FAB) *m/z* 1857.1 $(M + H)^+$.

TLC; Rf 0.36 (CH_2Cl_2-MeOH=9:1).

$[\alpha]_D^{20}$= –6.2 (c=1 in $CHCl_3$)

D-1-*O*-(1',2'-Di-*O*-heptanoyl-*sn*-glycero)phosphatidyl-*myo*-inositol-2,3,4,5,6-pentakisphosphate D-HIPPO [(-)-1]

To the solution of **(+)-10** (0.029 g, 0.016 mmol) in *t*-BuOH (8 ml) and H_2O, (1.5 ml) was added 10% Pd-C (0.032 g, 0.030 mmol), and the resulting mixture was stirred at room temperature under hydrogen for 24 h. The mixture was filtered through a pad of celite, and then celite pad was washed with H_2O. The resulting filtrate was lyophilized. The residue was dissolved in H_2O (2 ml), and filtered through cation-exchenge resin. To the resulting filtrate was added triethylamine (0.020 ml), and concentrated under reduced pressure. The resulting residue was lyophilized to afford **(–)-1** (0.013 g, 40%) as a white solid.

^{1}H NMR (D_2O) δ: 0.87 (6H, bs, CH_3 x 2), 1.22-1.31 (111H, m, CH_2 x 6, $NCH_2C\underline{H_3}$ x 33), 1.54-1.61 (4H, m, CH_2 x 2), 2.40 (2H, t, *J* = 7.1 Hz, CH_2), 2.42 (2H, t, *J* = 7.1 Hz, CH_2), 3.17-3.24 (66H, q, *J* = 7.1 Hz, $NC\underline{H_2}CH_3$ x 33), 4.34-4.82 (10H, m, $C\underline{H_2}CHC\underline{H_2}$, CH x 6), 5.34 (1H, bs, CH).

^{13}C NMR (D_2O + 1,4-dioxan) δ: 8.8, 13.9, 22.4, 24.9, 28.5, 31.3, 34.3, 34.4, 34.6, 39.3, 44.0, 47.3, 51.7, 63.6, 64.9, 65.8, 68.8, 71.6, 73.9, 76.0, 76.7, 78.0, 176.9, 177.2.

IR (Kbr) 3020, 2830, 2700, 2640, 2495, 1735, 1465, 1065 cm^{-1}.

HRMS(FAB) *m/z* calcd for $C_{23}H_{47}O_{28}P_6$ $(M - H)^-$ 957.0597 Found: 957.0782.

$[\alpha]_D^{20}$= –4.5 (c=0.5 in H_2O)

L-3-*O*-(1',2'-Di-*O*-heptanoyl-*sn*-glycero)phosphatidyl-*myo*-inositol-1,2,4,5,6- pentakisphosphate L-HIPPO [(+)-2]

(–)-11 (0.93 g, 0.050 mmol) was allowed to react, under the same conditions as described for the preparation of **(–)-1**, to give **(+)-2** (0.044 g, 43%) as a colorless oil.

^{1}H NMR (D_2O) δ: 0.82-0.84 (6H, m, CH_3 x 2), 1.26 (75H, bs, CH_2 x 6, CH_3 x 21), 1.54-1.59 (4H, m, CH_2 x 2), 2.37 (2H, t, *J* = 7.3 Hz, CH_2), 2.41 (2H, t, *J* = 7.0 Hz, CH_2), 3.18 (42H, bs, CH_2 x 21), 4.10-4.58 (9H, m, $C\underline{H_2}CHC\underline{H_2}$, CH x 5), 4.92-4.95 (1H, m, CH), 5.30 (1H, bs, CH).

^{13}C NMR (D_2O + 1,4-dioxan) δ: 8.9, 14.0, 22.5, 24.9, 28.5, 31.3, 31.4, 34.5, 34.6, 47.3, 63.5, 64.8, 71.5, 71.6, 73.8, 73.9, 76.0, 76.3, 76.8, 76.9, 78.0176.8, 177.2.

IR (Kbr) 3440, 2940, 2730, 2670, 1735, 1465, 1065 cm^{-1}.

HRMS(FAB) *m/z* calcd for $C_{23}H_{47}O_{28}P_6$ $(M - H)^-$ 957.0597 Found: 957.0638.

$[\alpha]_D^{20}$= 1.4 (c=0.5 in H_2O)

1 Mills, S. J. & Potter B. V. L. Synthesis of the enantiomers of *myo*-inositol 1,2,4,5-tetrakis-phosphate, a regioisomer of myo-inositol 1,3,4,5-tetrakisphosphate. *Journal of the Chemical Society, Perkin Transactions 1*, 1279-1286 (1997).

2 Nagashima, N. & Ohno, M. An Efficient *O*-Monoalkylation of Dimethyl L-Tartrate via *O*-Stannylene Acetal with Alkyl Halides in the Presence of Cesium Fluoride. *Chemistry Letters* **16**, 141-144 (1987).

3 Liu, C. & Potter, B. L. Synthesis of 3-Position-Modified Analogues of *myo*-Inositol 1,4,5-Trisphosphate, Tools for Investigation of the Polyphosphoinositide Pathway of Cellular Signaling. *The Journal of Organic chemistry* **62**. 8835-8340 (1997).

4 Oikawa, Y. *et al*. Selective hydrogenolysis of the benzyl protecting group for hydroxy function with raney nickel in the presence of the MPM (4-methoxybenzyl) and DMPM (3,4-dimethoxybenzyl) protecting groups. *Tetrahedron Letters* **25**. 5397-5400 (1984).

5 Classon, B *et al*. The p-Methoxybenzyl Group as Protective Group of the Anomeric Centre. Selective Conversions of Hydroxy Groups into Bromo Groups in p-Methoxybenzyl 2-Deoxy-2-phthalimido-beta-D-glucopyranoside. *Acta Chemica Scandinavica* **38b**. 419–422 (1984).

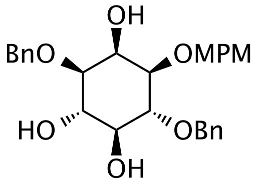
OH
BnO
OMPM
HO
OBn
OH

(–)-**4**

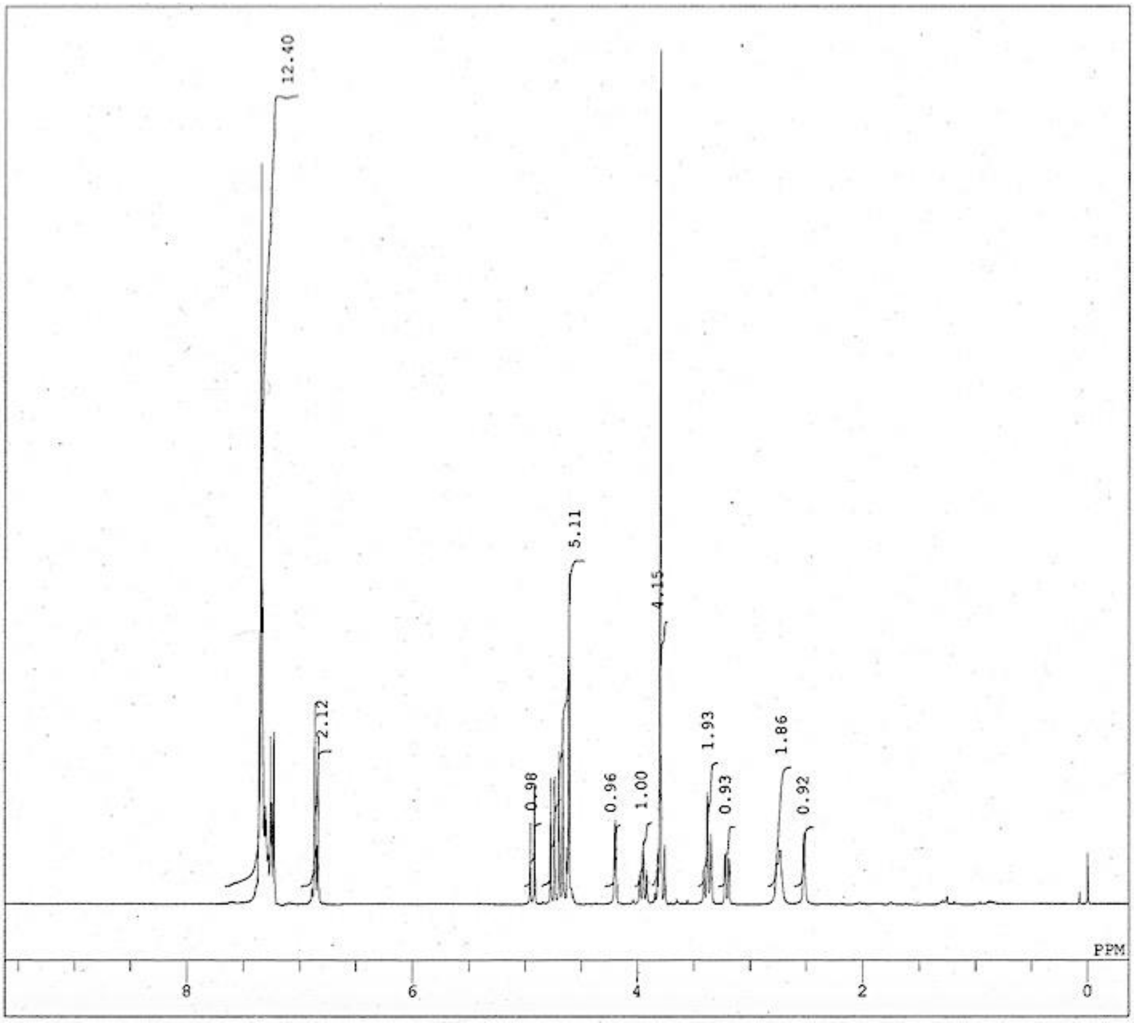
12.40
2.12
0.98
5.11
0.96
1.00
4.15
1.93
0.93
1.86
0.92
PPM
8
6
4
2
0

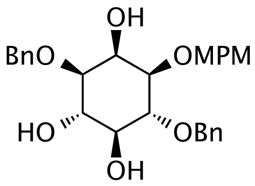

(–)-**4**

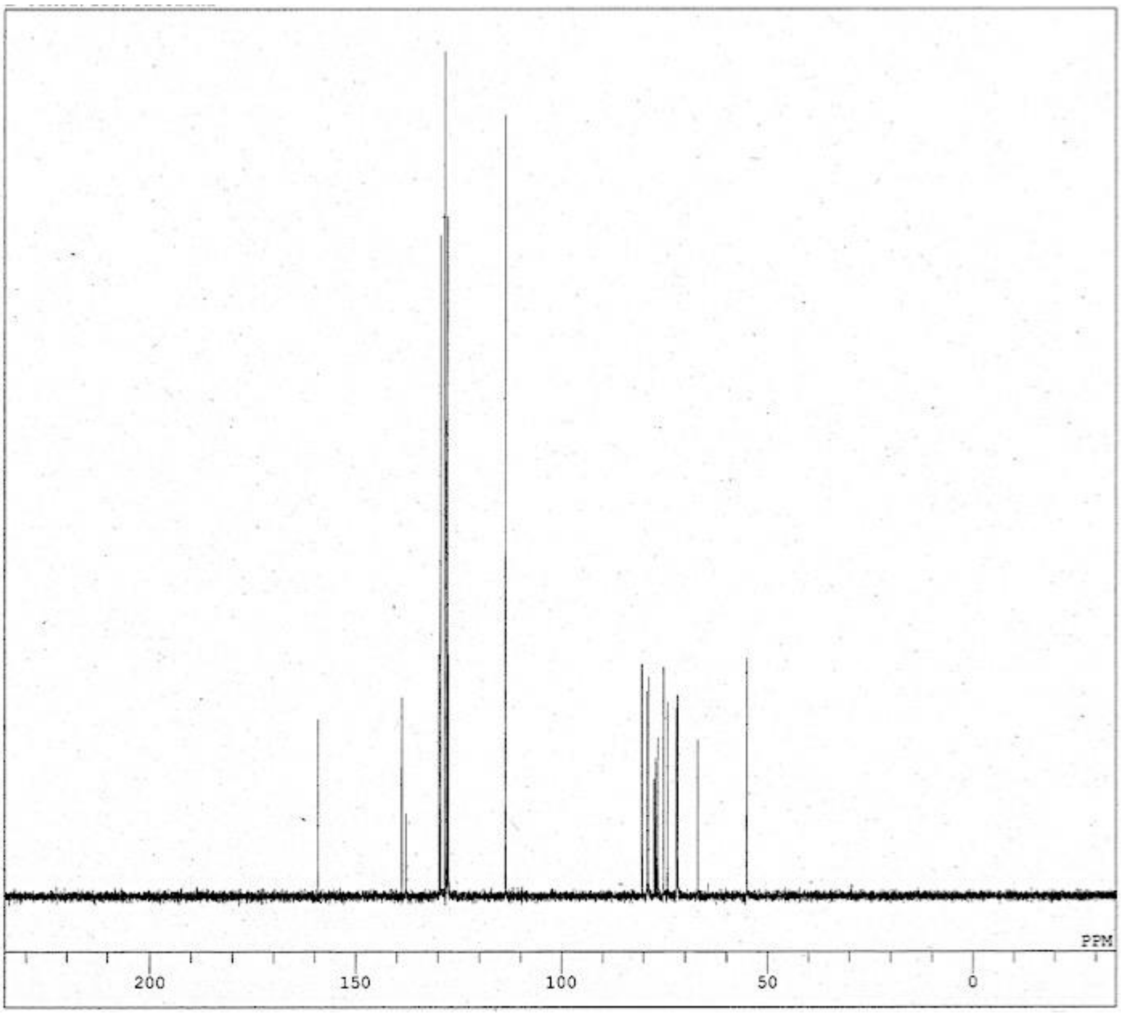

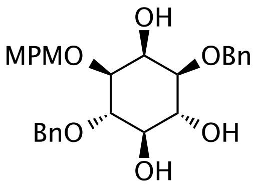
OH
MPMO
OBn
BnO
OH
OH

(+)-**4**

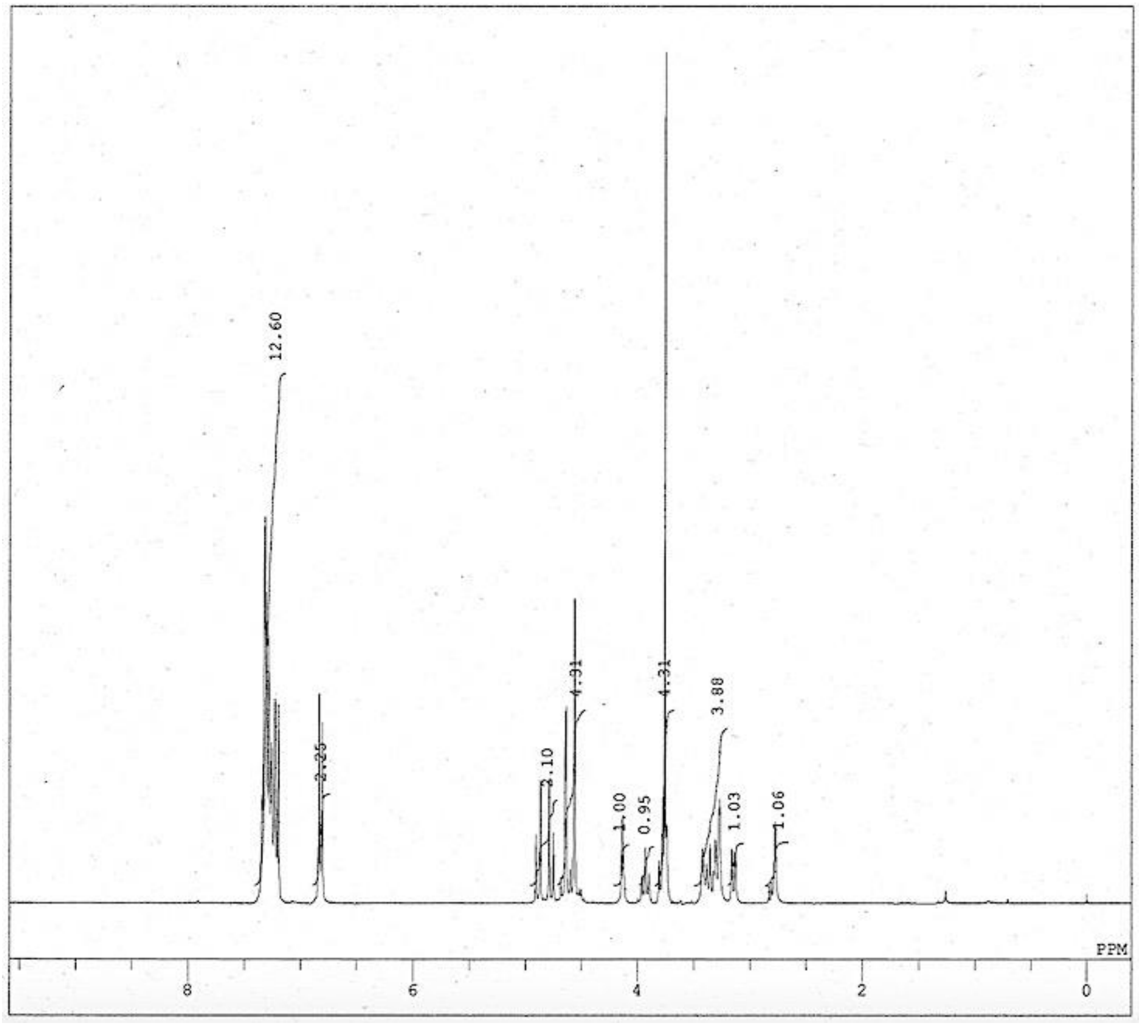
12.60
2.25
2.10
4.91
1.00
0.95
4.31
3.88
1.03
1.06
PPM
8
6
4
2
0

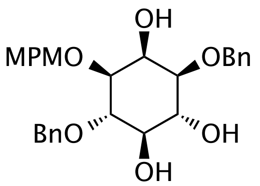

(+)-**4**

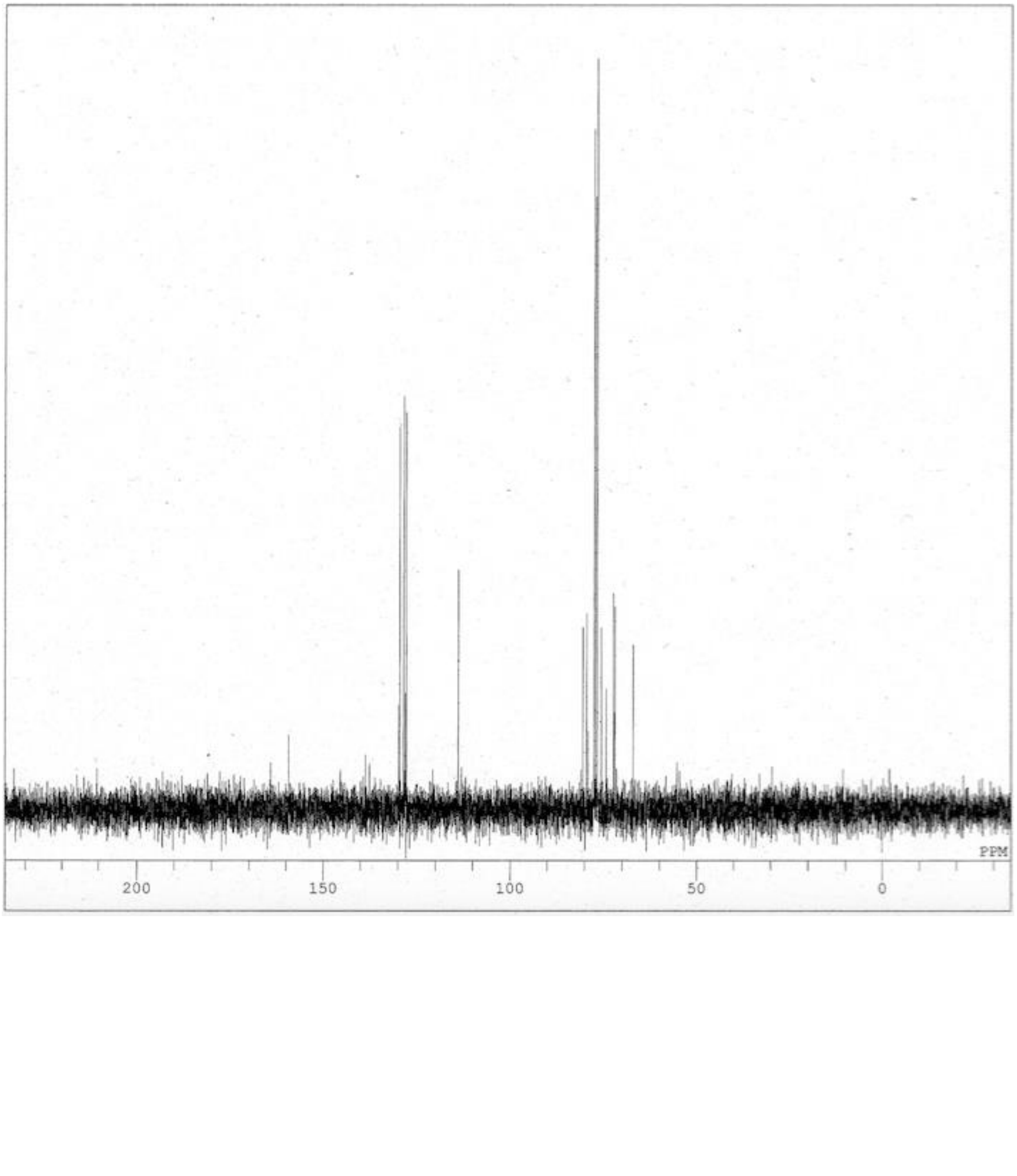

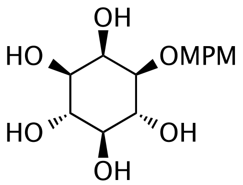
OH
HO
OMPM
HO
OH
OH

(–)-**5**

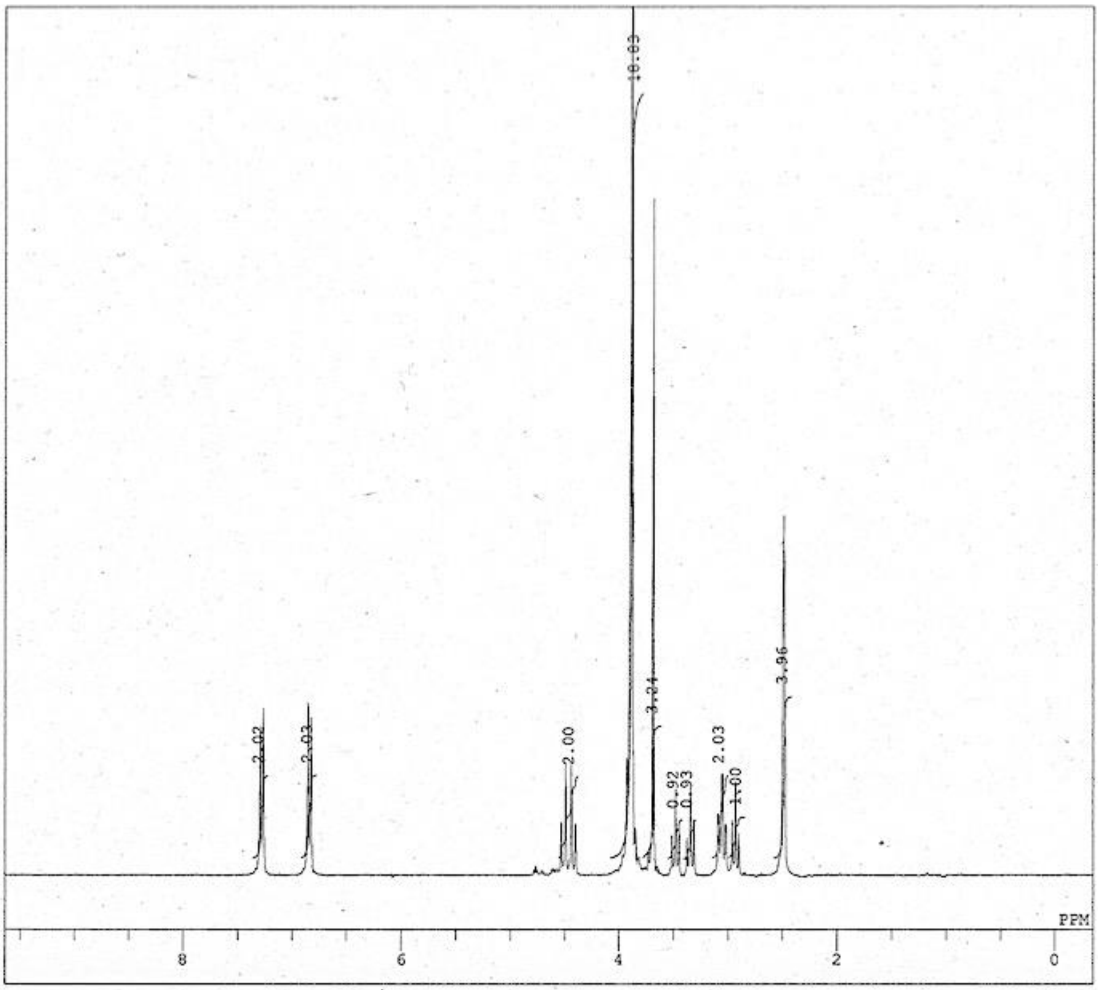
10.03
3.04
2.02
2.03
2.00
0.92
0.93
2.03
1.00
3.96
PPM
8
6
4
2
0

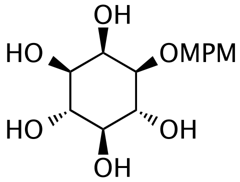

(–)-**5**

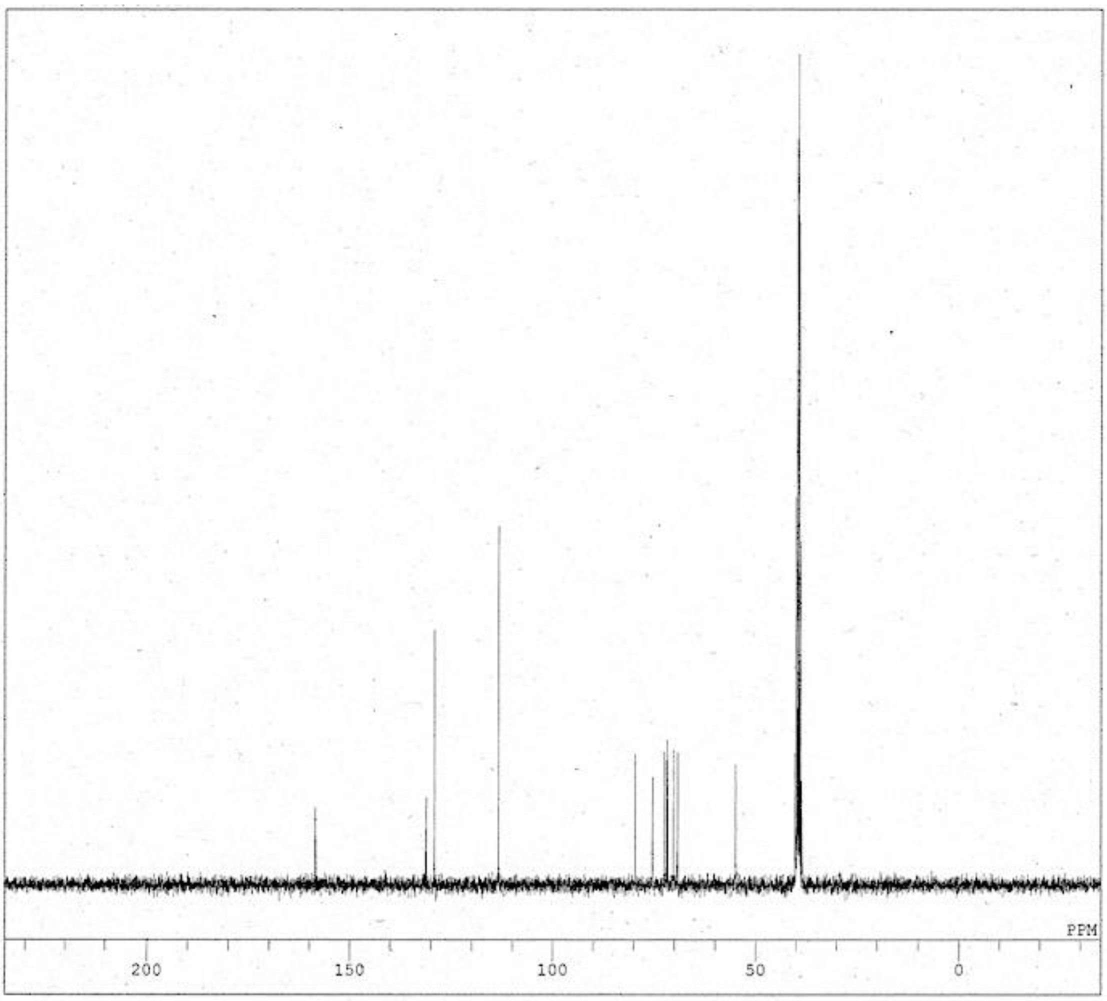

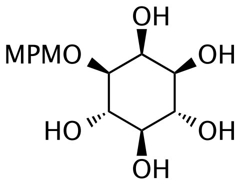
OH
MPMO
OH
HO
OH
OH
(+)-**5**

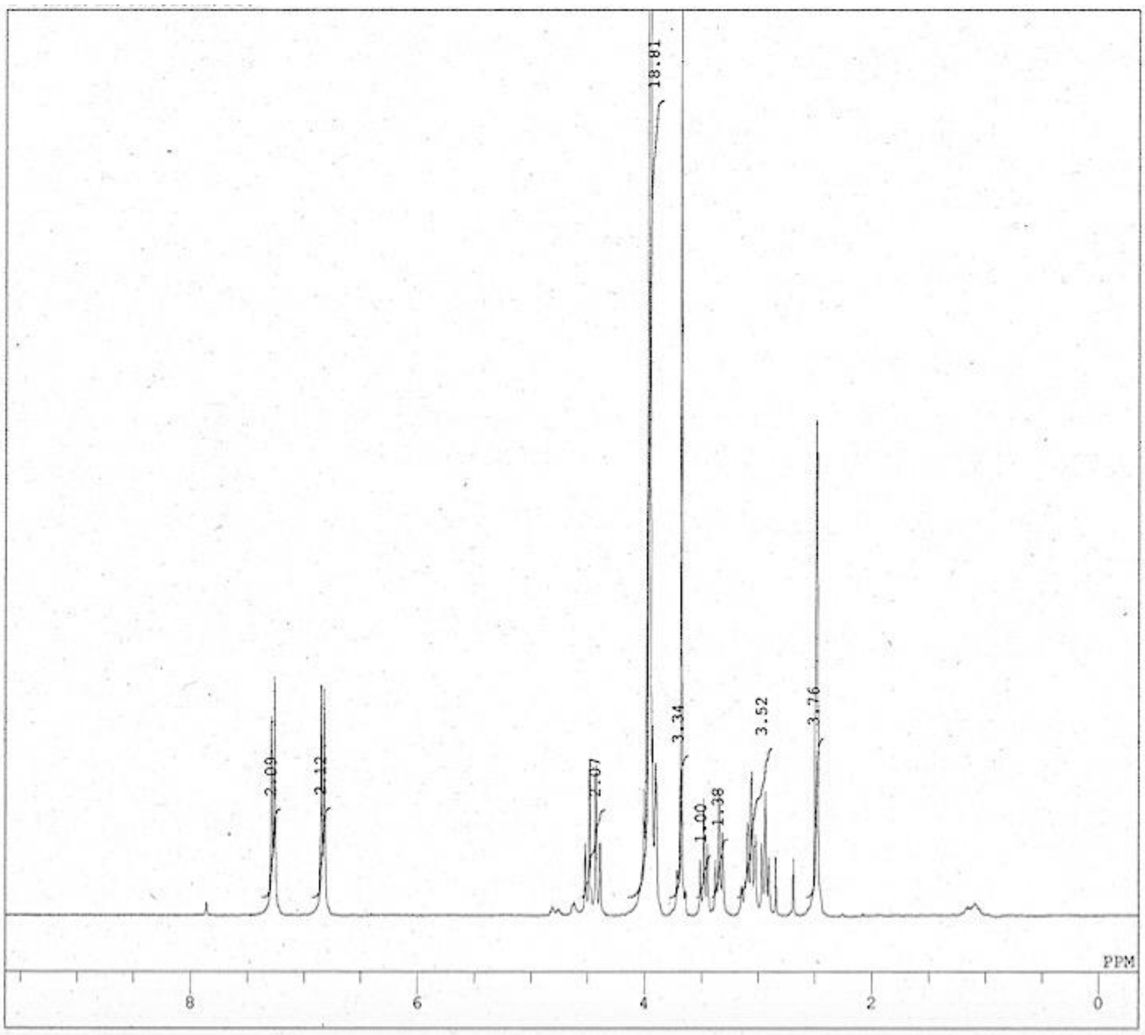
18.81
3.34
3.52
3.76
2.09
2.12
2.07
1.00
1.38
PPM
8
6
4
2
0

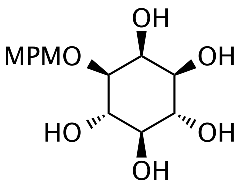

(+)-**5**

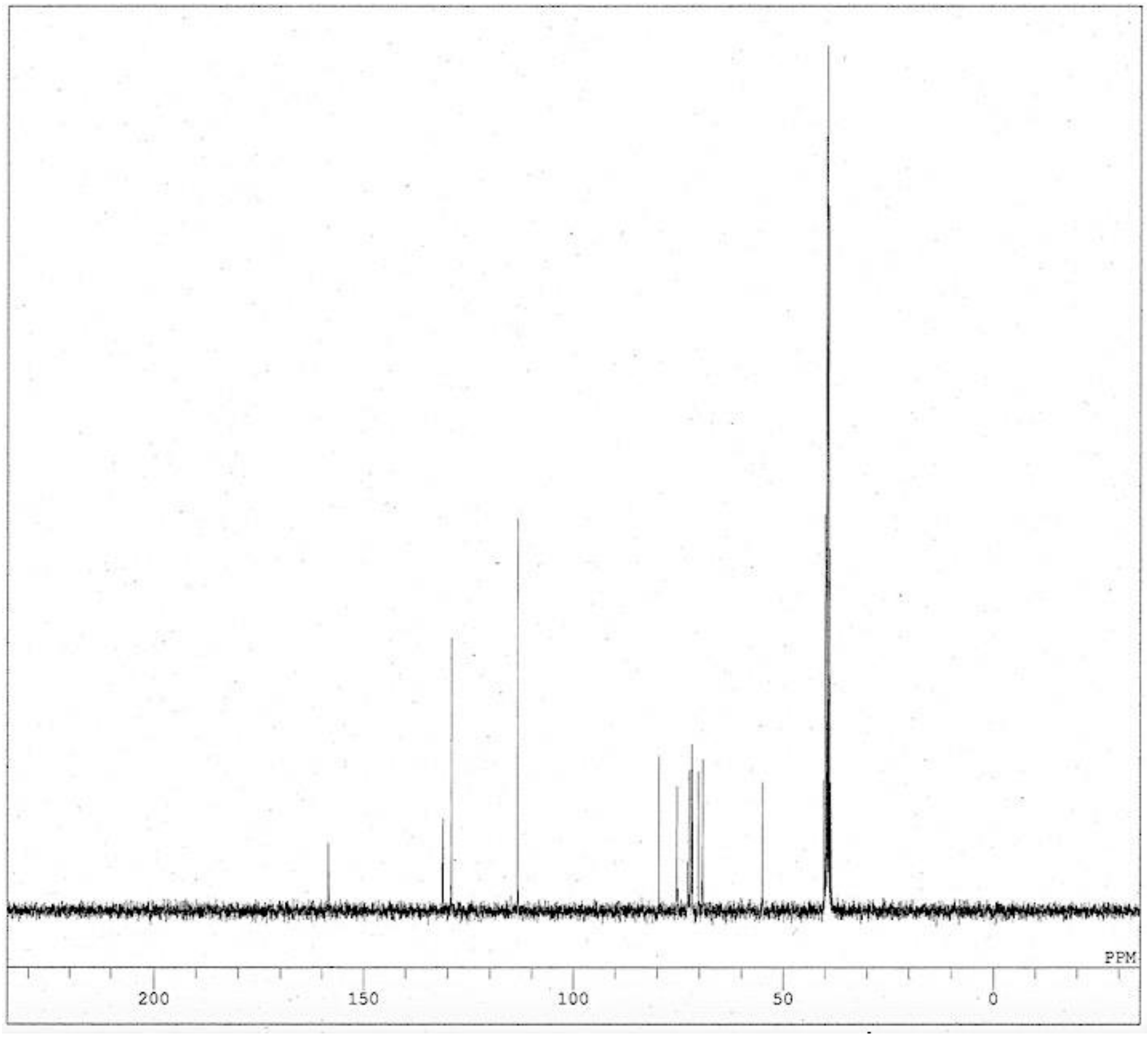

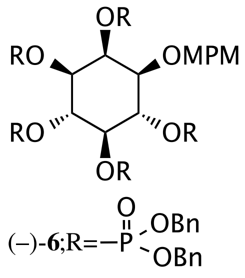
OR
RO
OMPM
RO
OR
OR
O
OBn
(–)-6;R=
P
OBn

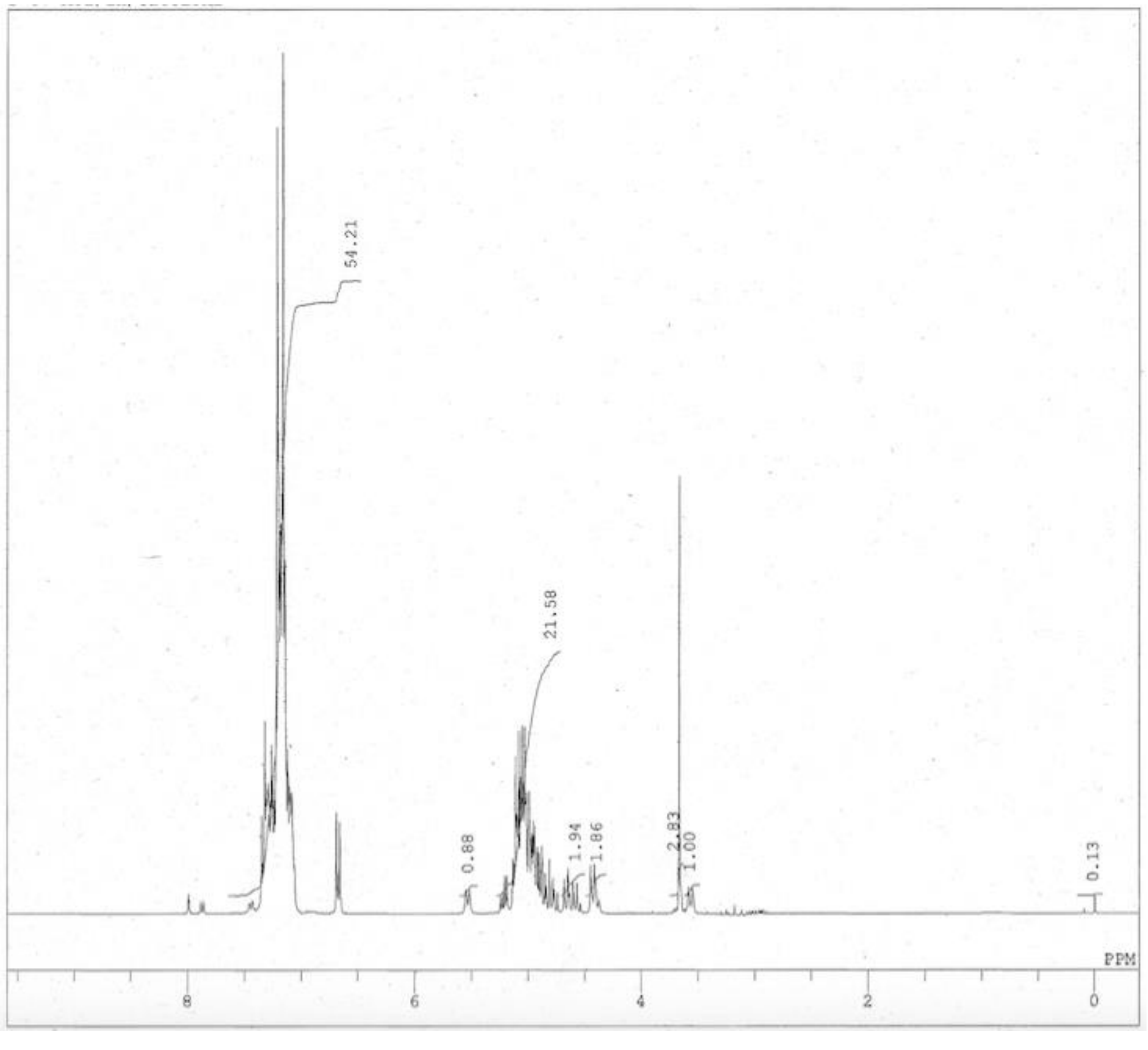
54.21
21.58
0.88
1.94
1.86
2.83
1.00
0.13
PPM
8
6
4
2
0

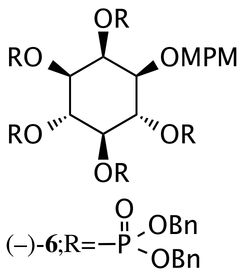

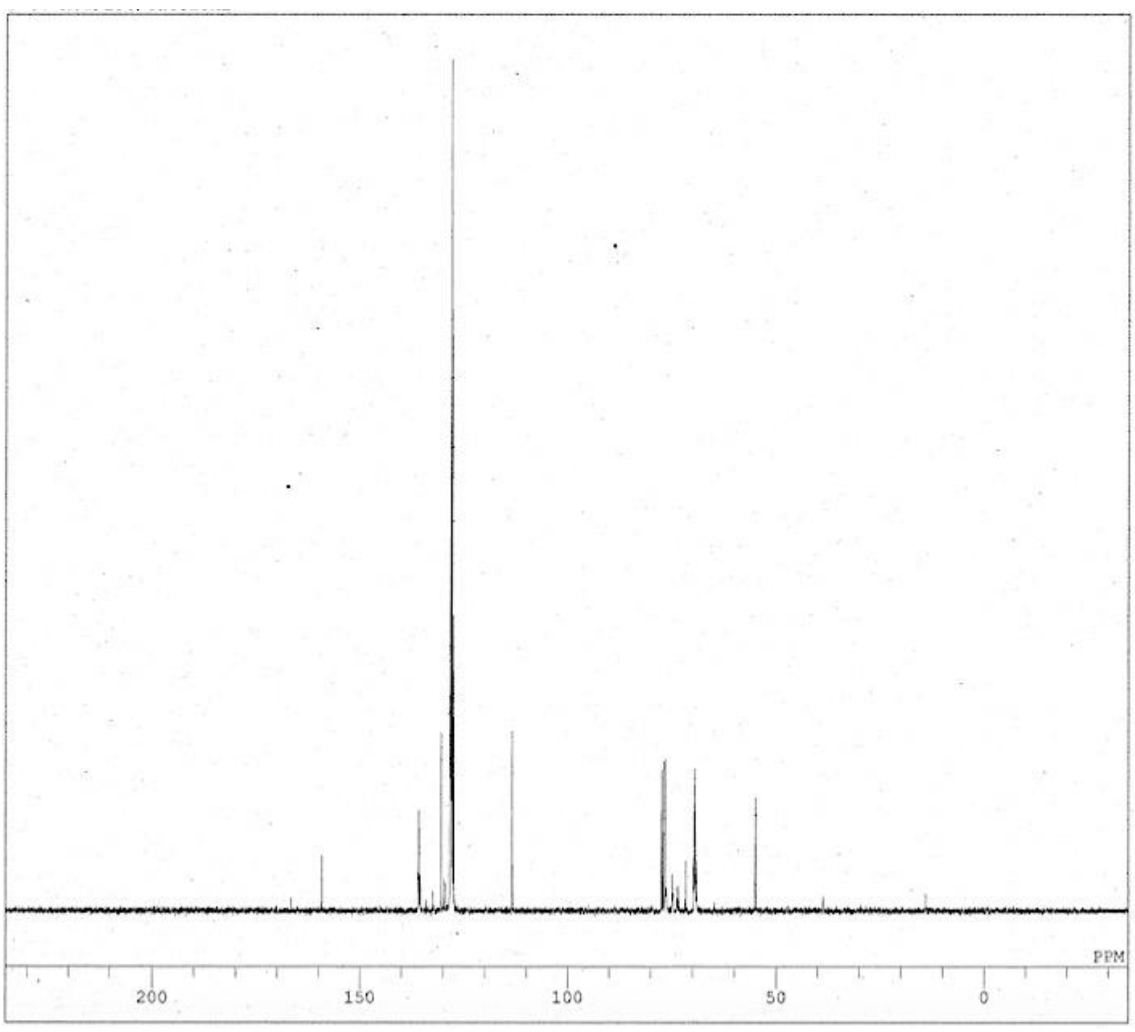
PPM
200
150
100
50
0

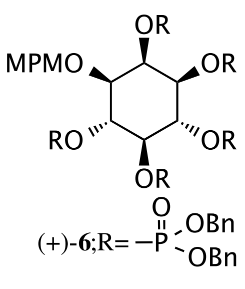
MPMO
OR
OR
RO
OR
OR
O
OBn
(+)-6;R= –P
OBn

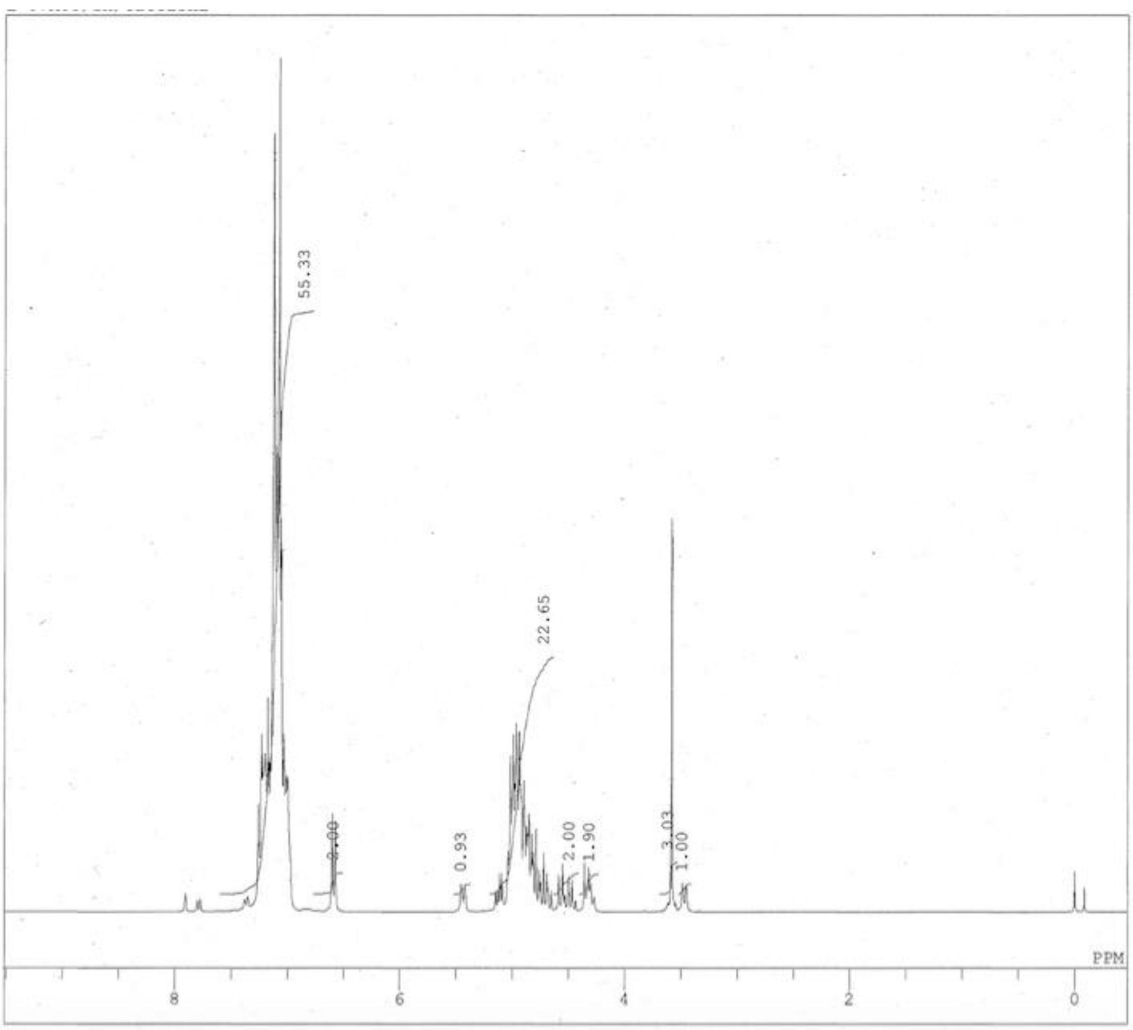
55.33
22.65
2.00
0.93
2.00
1.90
3.03
1.00
PPM
8
6
4
2
0

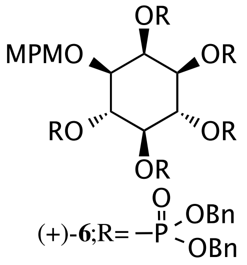
OR
MPMO
OR
RO
OR
OR
(+)-**6**;R=
O
OBn
P
OBn

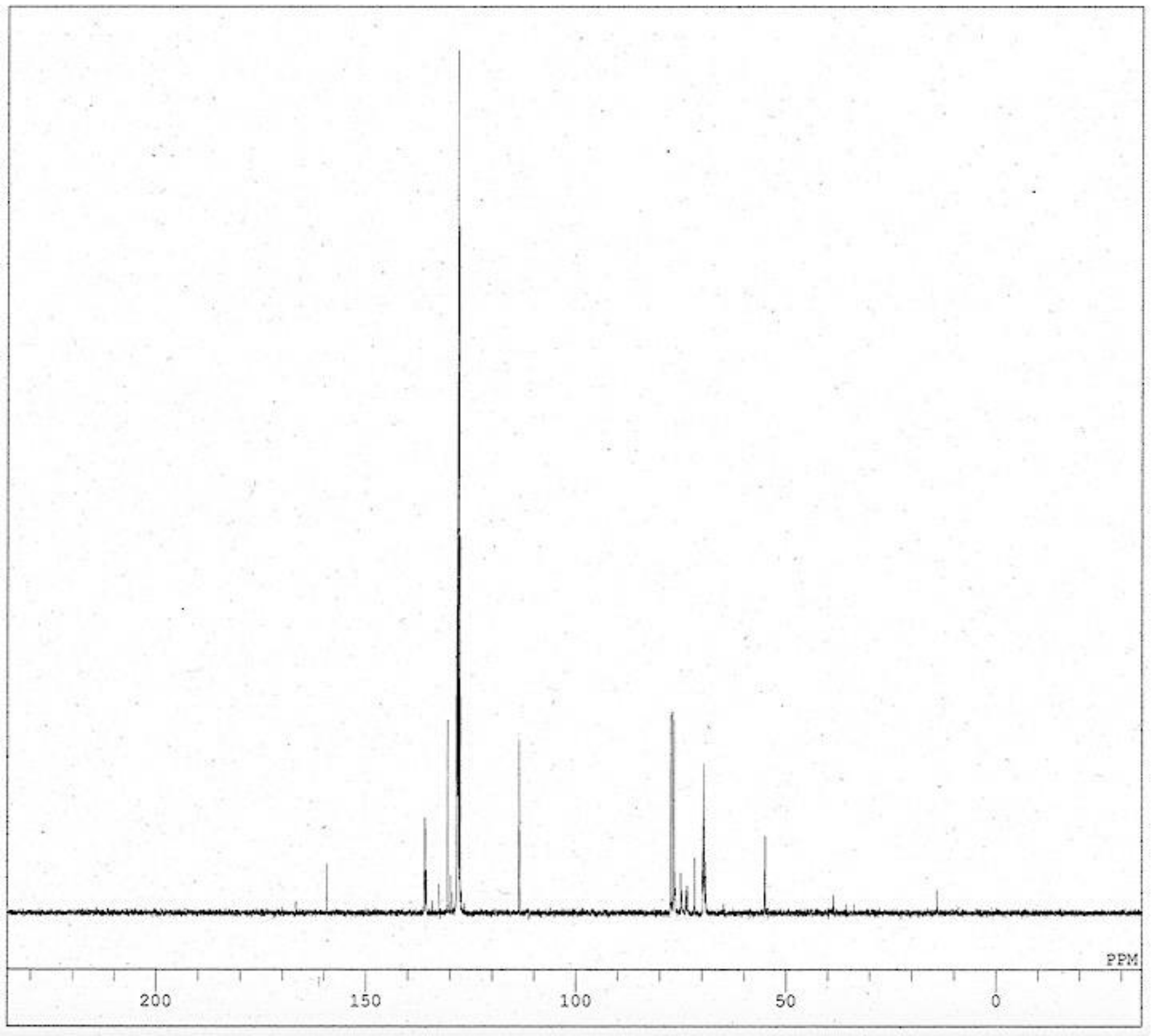
PPM
200
150
100
50
0

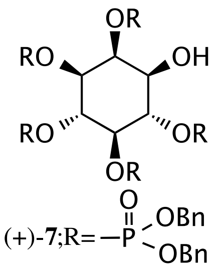
OR
RO
OH
RO
OR
OR
(+)-7;R=
O
OBn
P
OBn

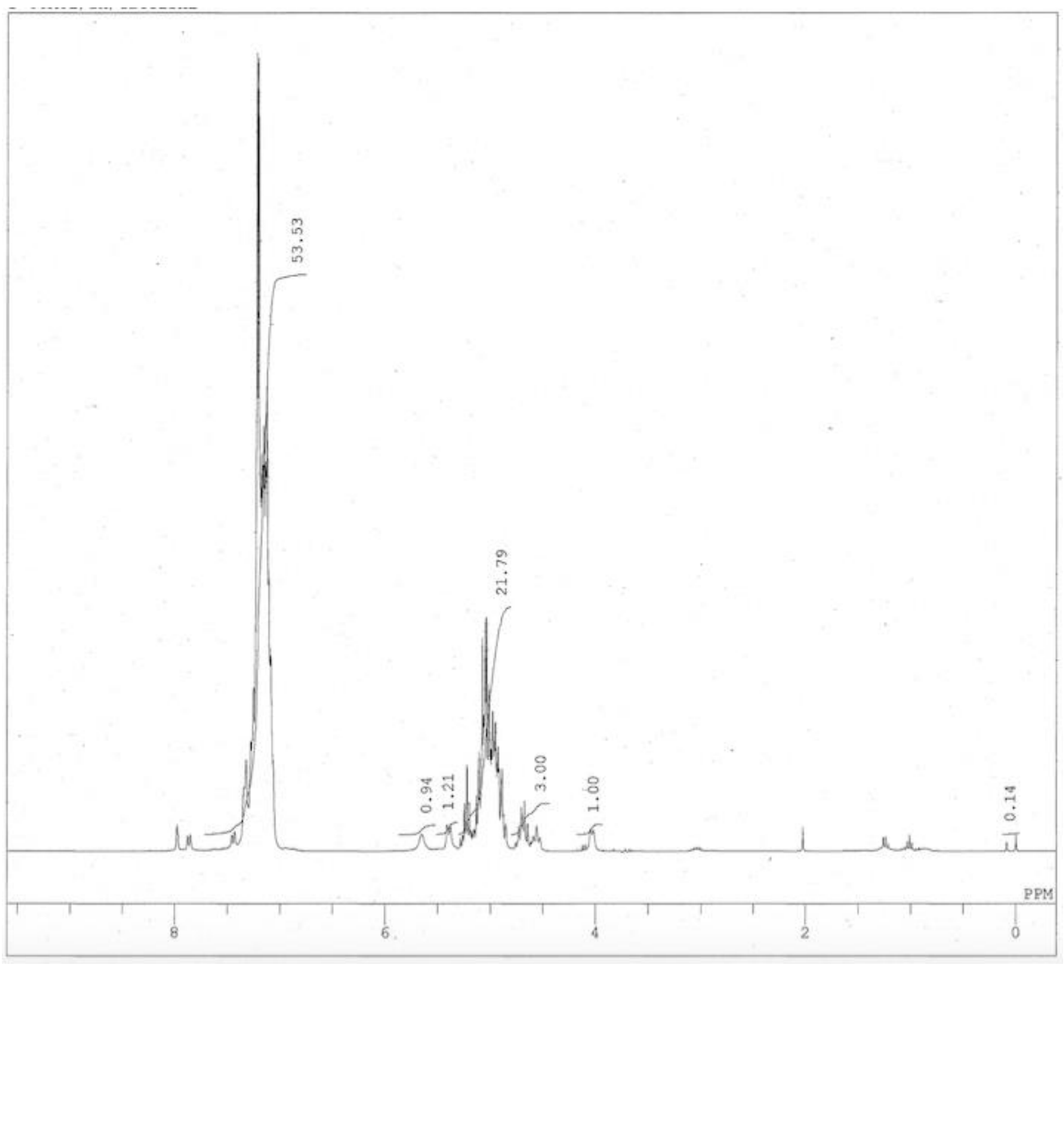
53.53
21.79
0.94
1.21
3.00
1.00
0.14
PPM
8
6
4
2
0

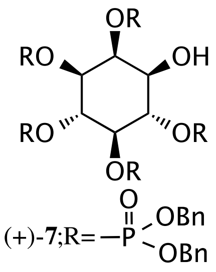
OR
RO
OH
RO
OR
OR
O
OBn
(+)-7;R=
P
OBn

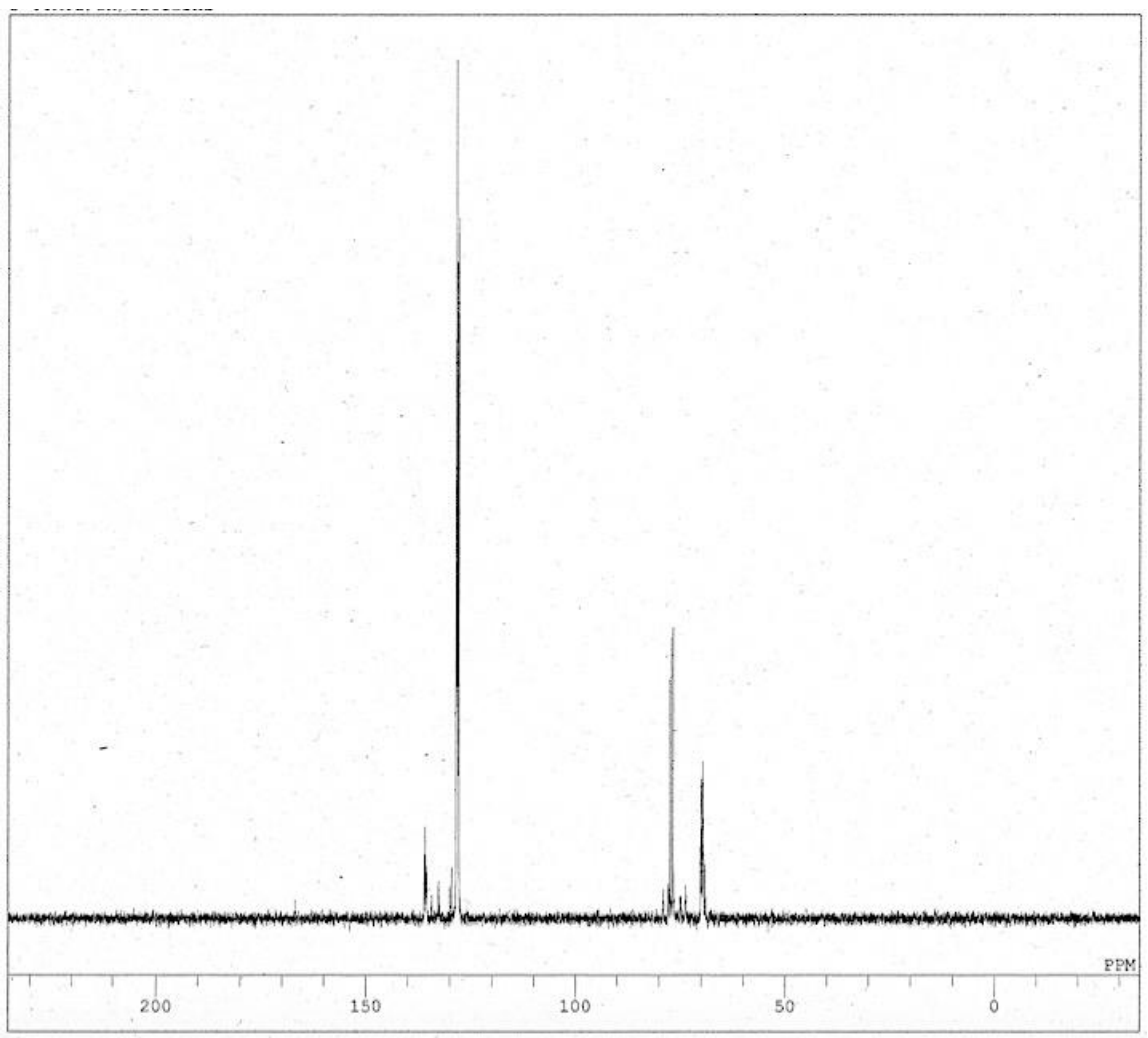
PPM
200
150
100
50
0

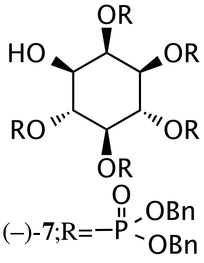
OR
HO
OR
RO
OR
OR
O
OBn
(−)-7;R=
P
OBn

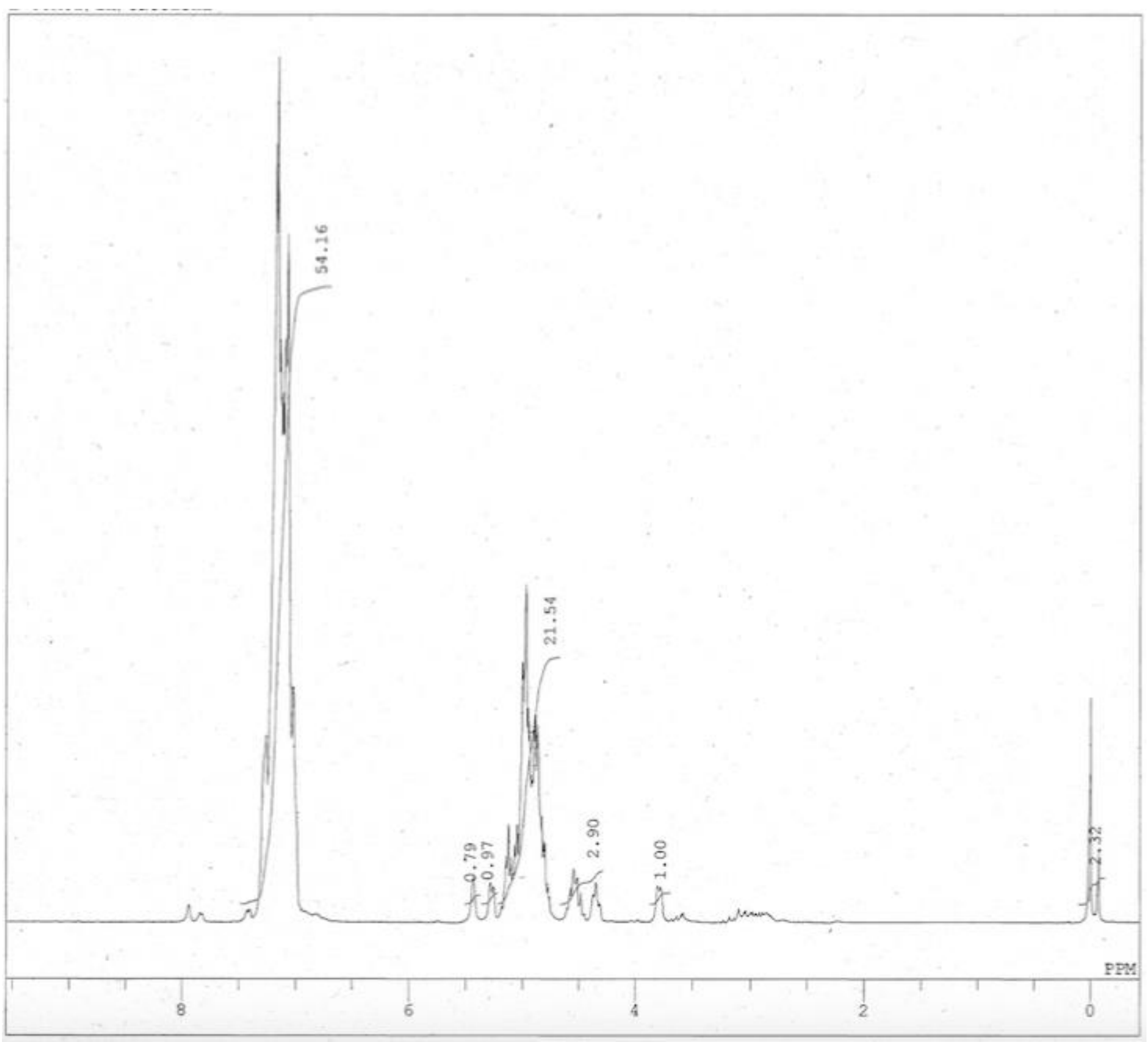
54.16
21.54
0.79
0.97
2.90
1.00
2.32
PPM
8
6
4
2
0

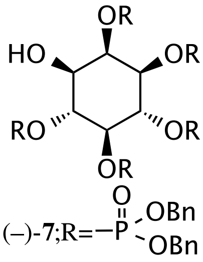

(–)-7;R=

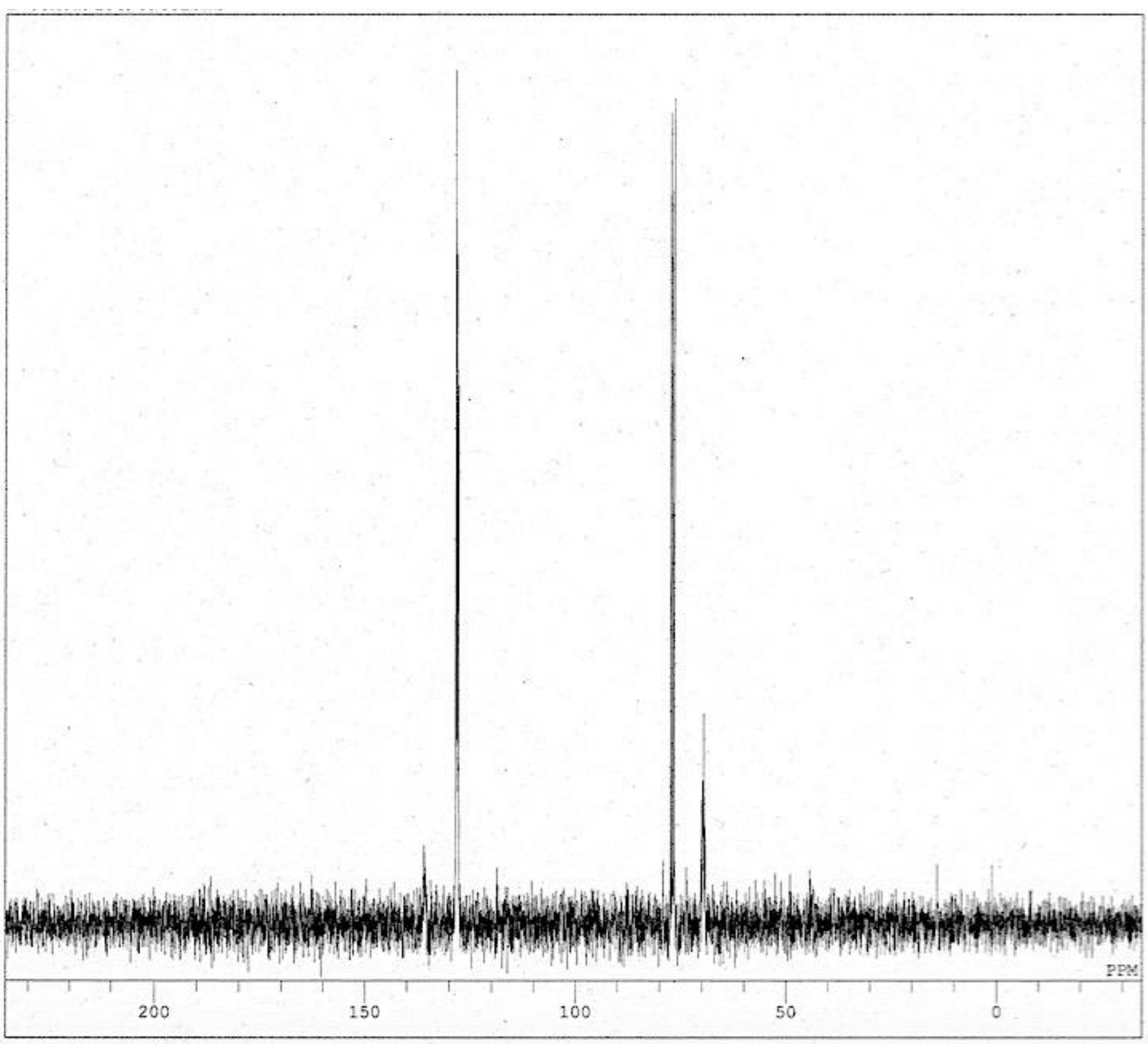

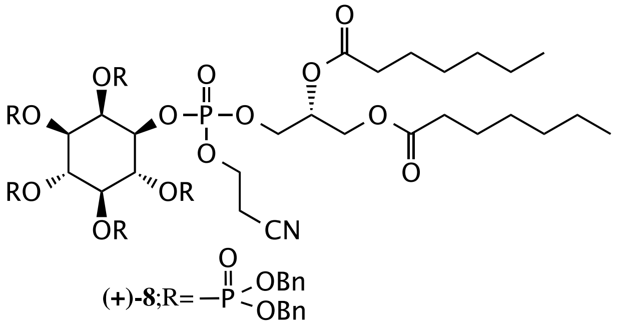
OR
RO
O–P–O
O
O
RO
OR
OR
CN
O
O
O
O
(+)-8;R=
O
P
OBn
OBn

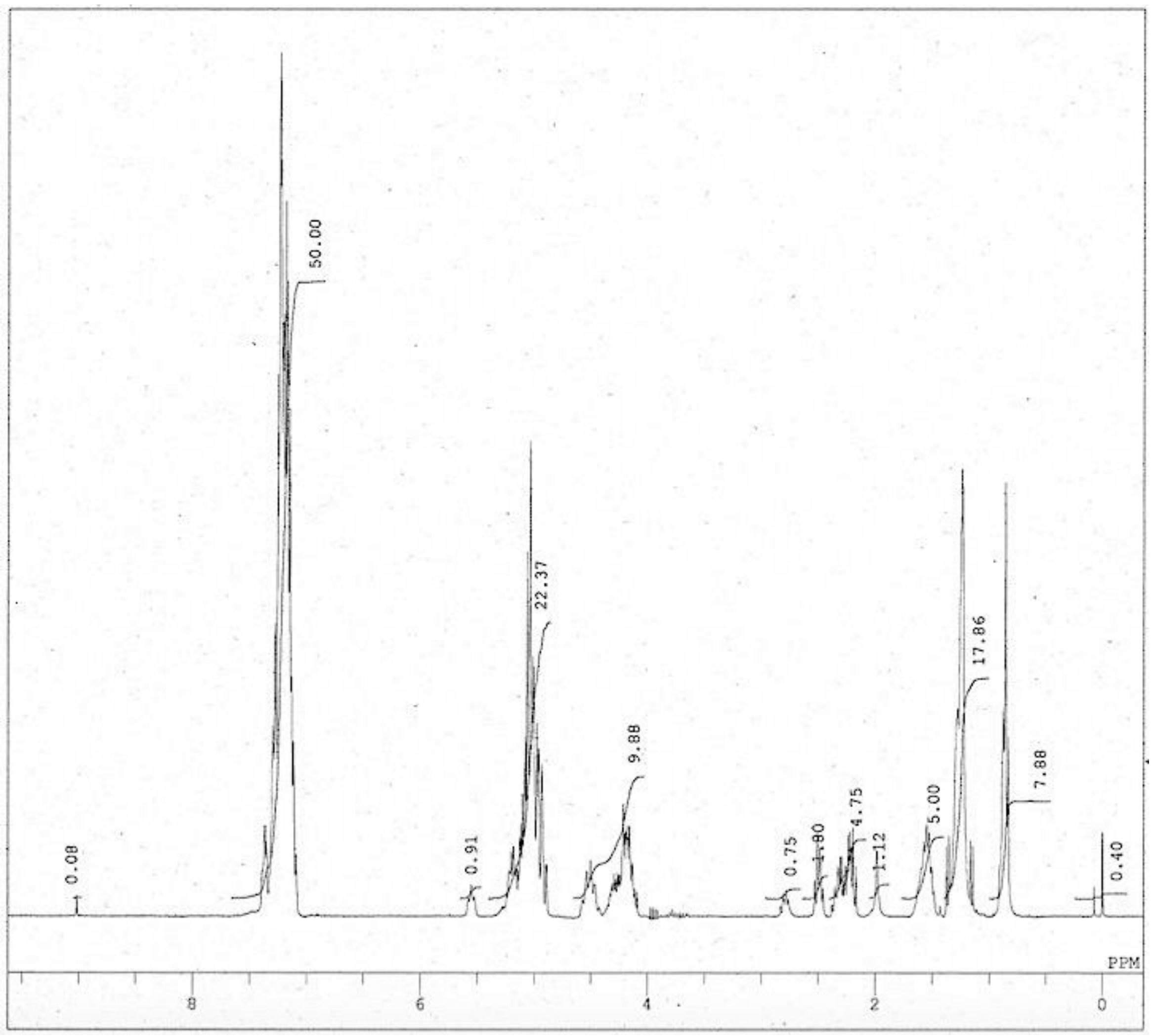
0.08
50.00
0.91
22.37
9.88
0.75
1.80
4.75
1.12
5.00
17.86
7.88
0.40
PPM
8
6
4
2
0

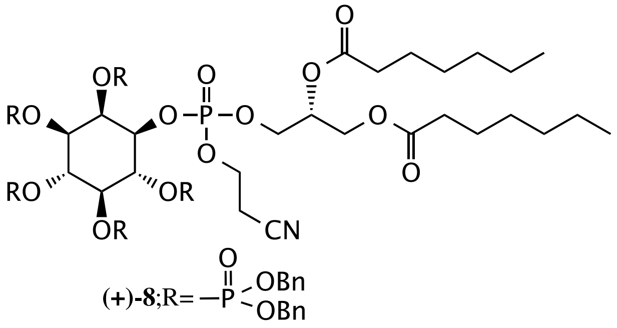
OR
RO
RO
OR
OR
O
P
O
O
O
CN
O
O
O
O
(+)-8;R=
P
OBn
OBn

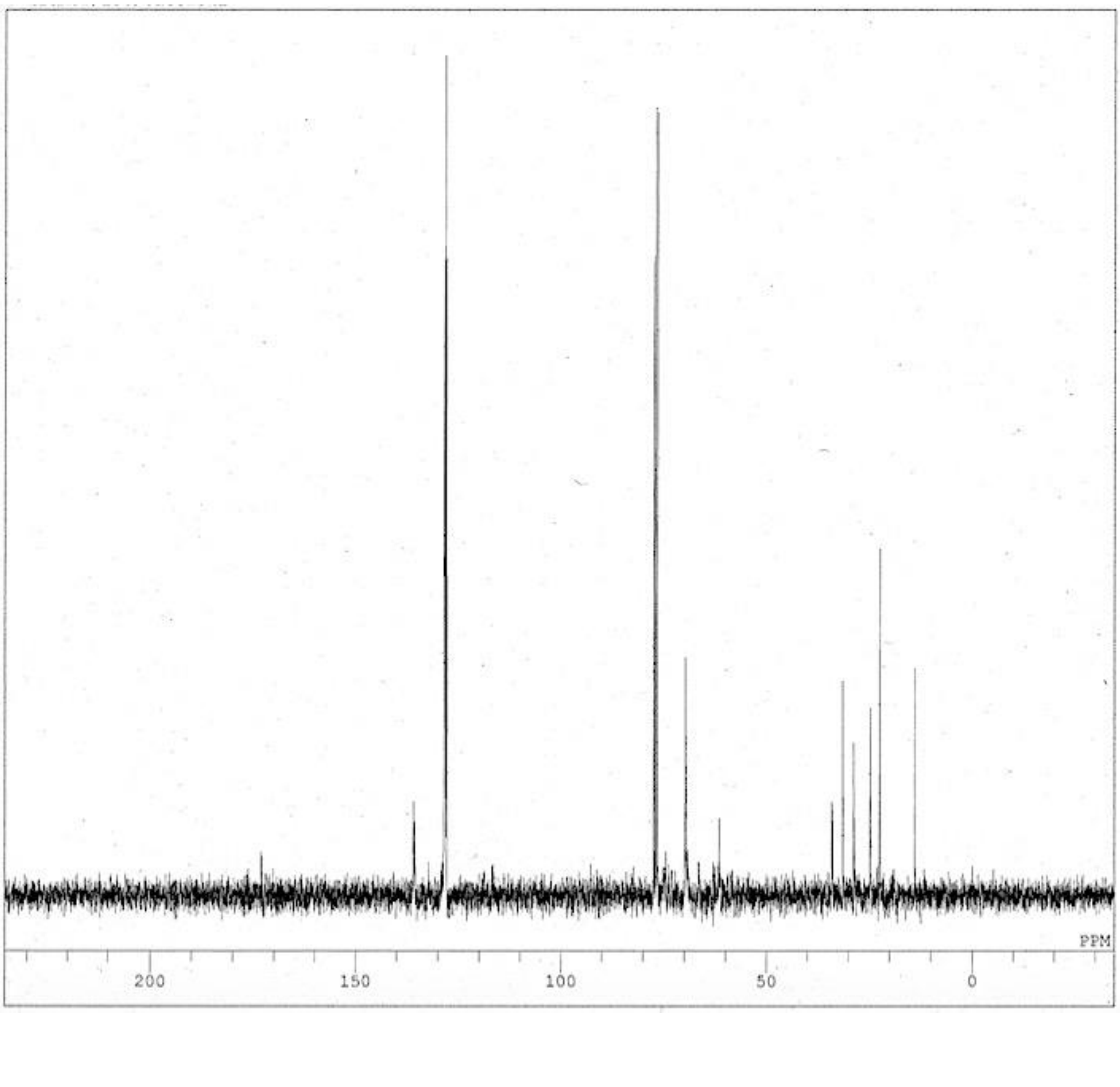
PPM
200
150
100
50
0

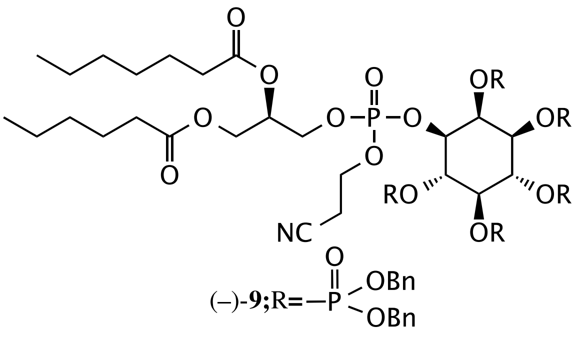
(–)-9; R=
OBn
OBn
OR
OR
RO
OR
OR
NC

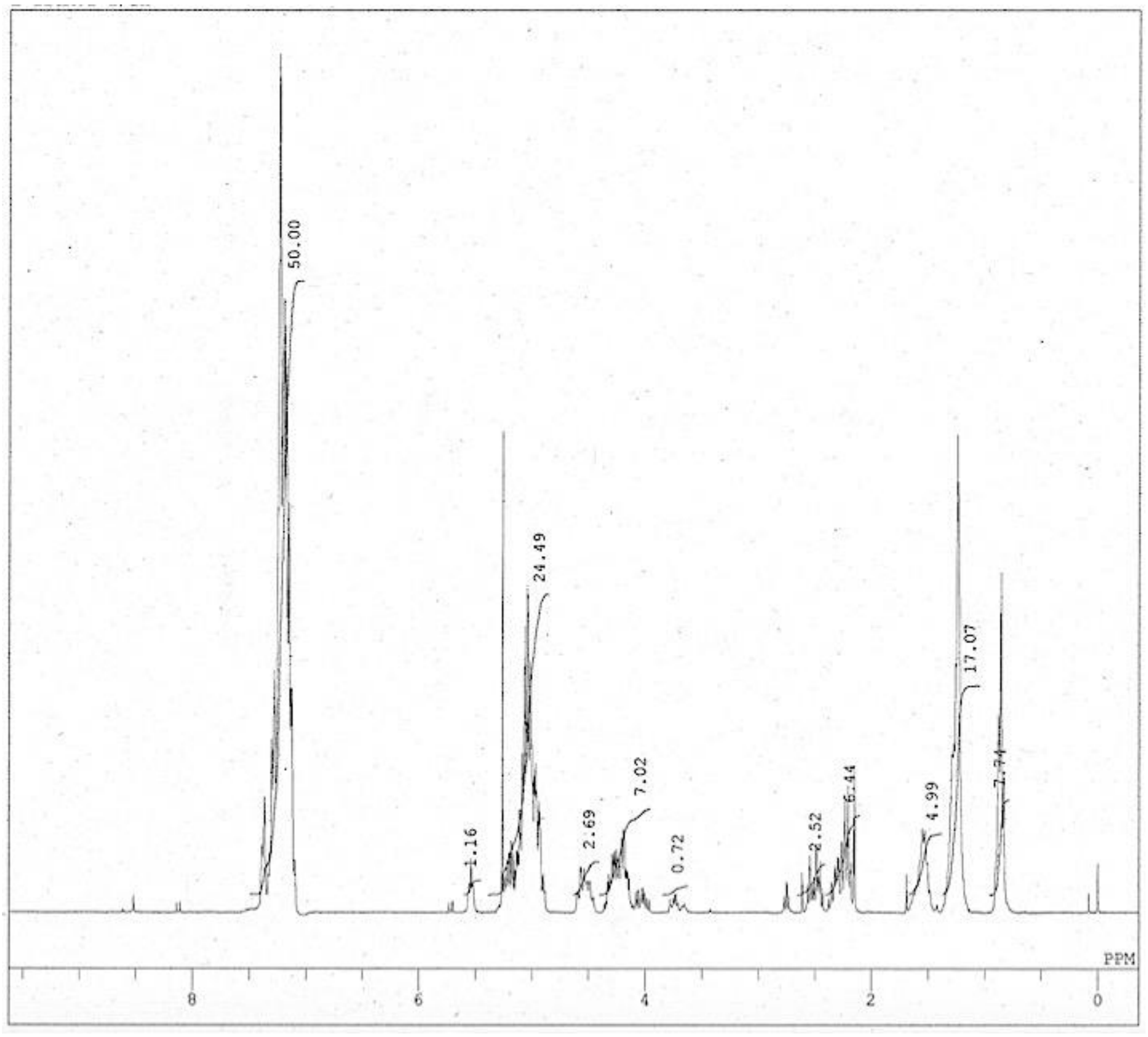
50.00
24.49
17.07
7.74
7.02
6.44
4.99
2.69
2.52
1.16
0.72
PPM
8
6
4
2
0

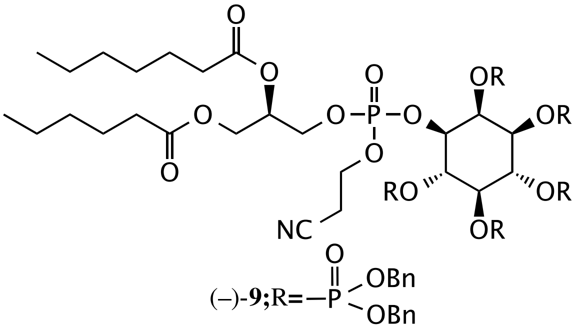
(–)-9;R=
OBn
OBn
OR
RO
NC

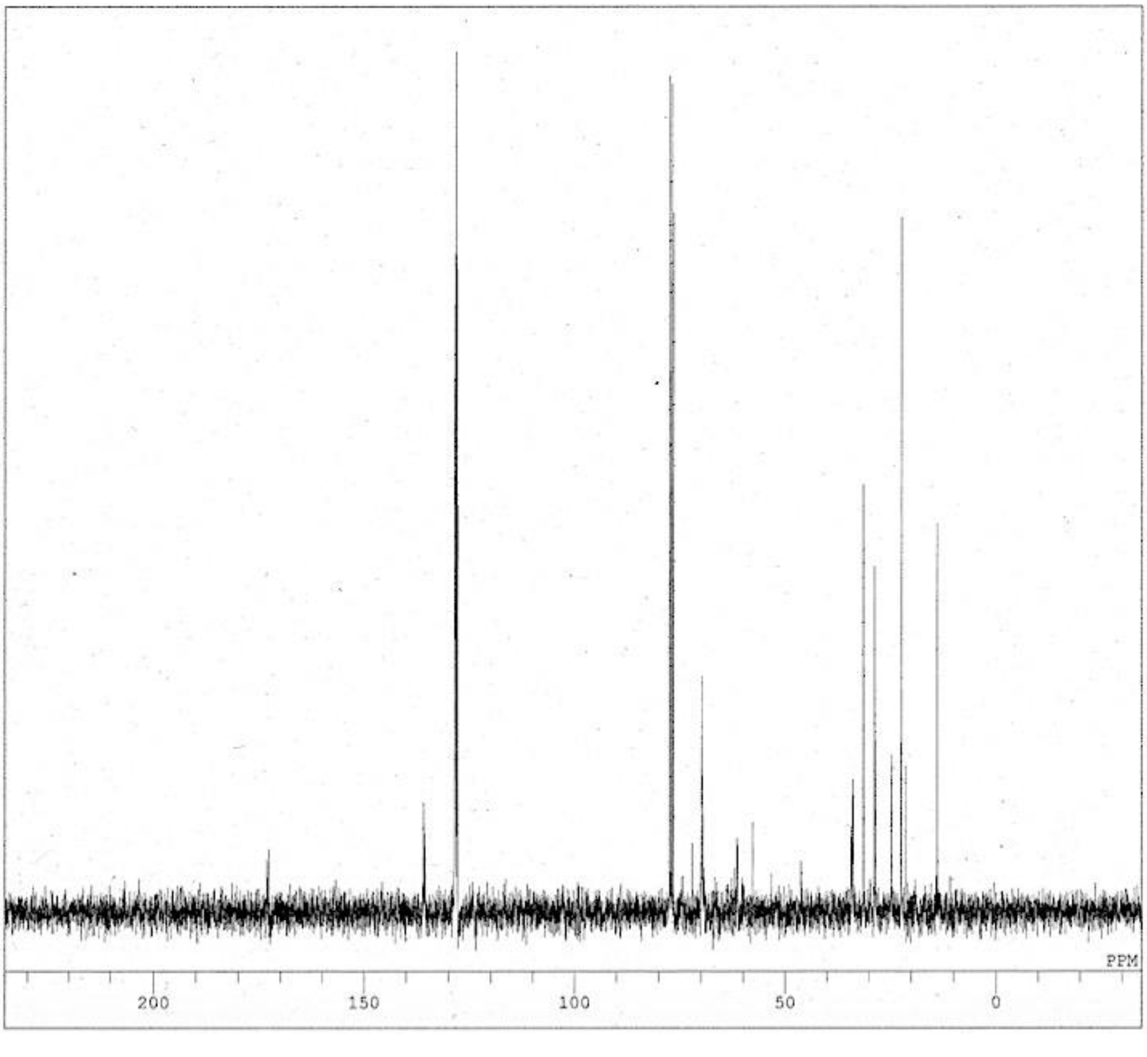
PPM
200
150
100
50
0

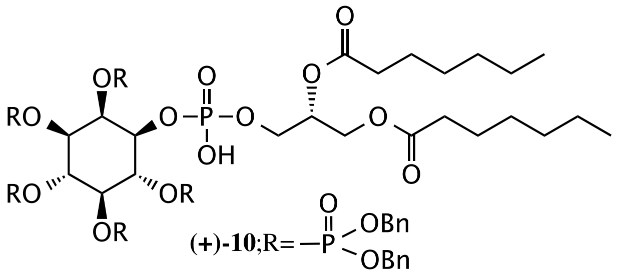
(+)-10;R=
OBn
OBn

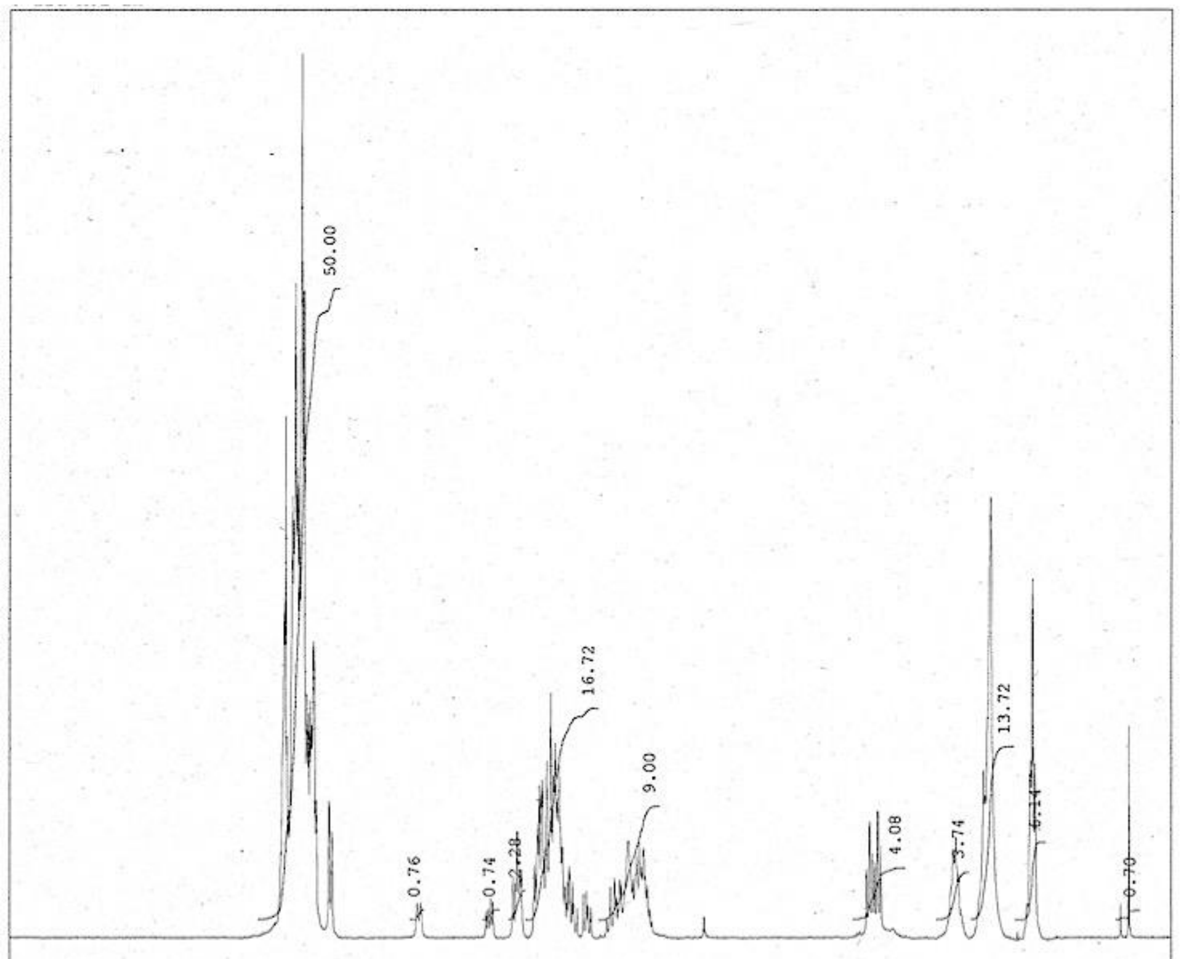
50.00
16.72
9.00
0.76
0.74
4.08
3.74
13.72
6.14
0.70
PPM
8
6
4
2
0

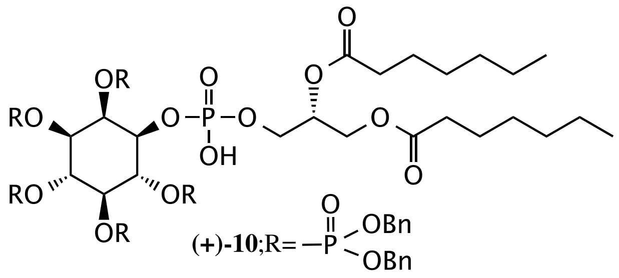
(+)-10;R=

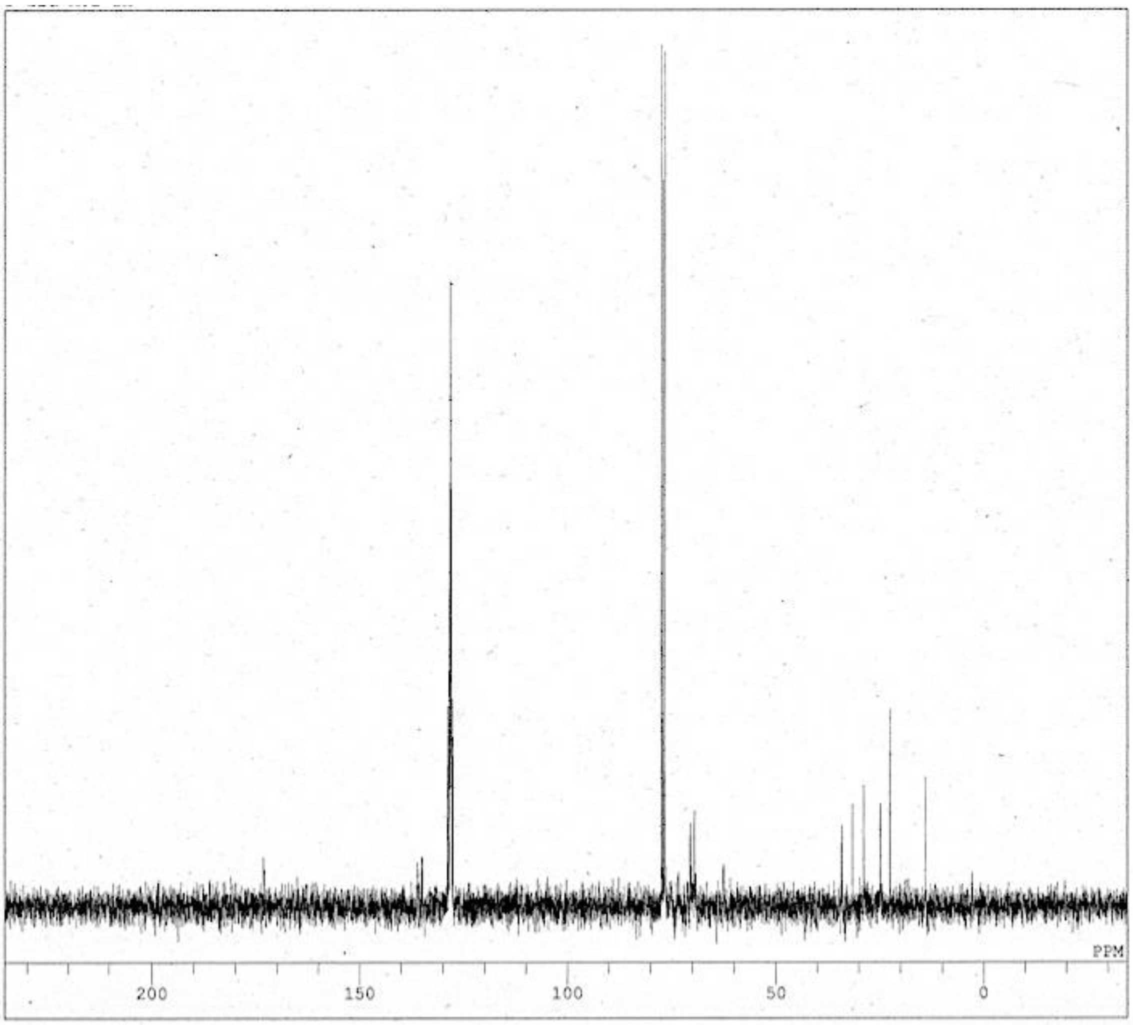
PPM
200
150
100
50
0

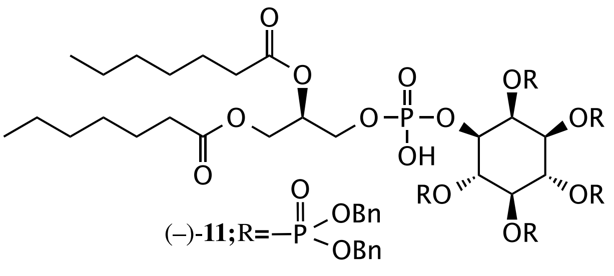

O
O
O
O-P-O
OH
OR
OR
OR
RO
OR
OR
O
(–)-11;R=–P
OBn
OBn

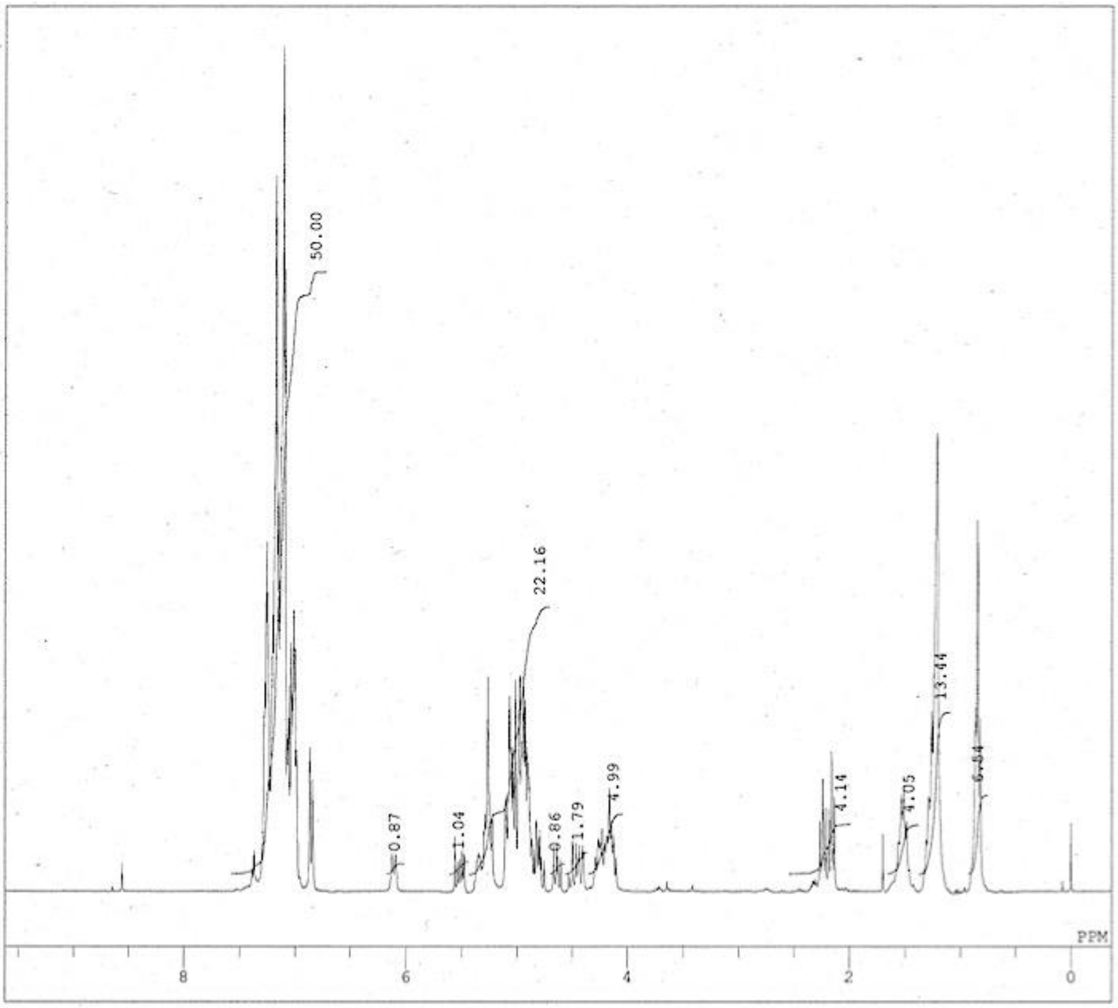

50.00
22.16
0.87
1.04
0.86
1.79
4.99
4.14
4.05
13.44
6.84
PPM
8
6
4
2
0

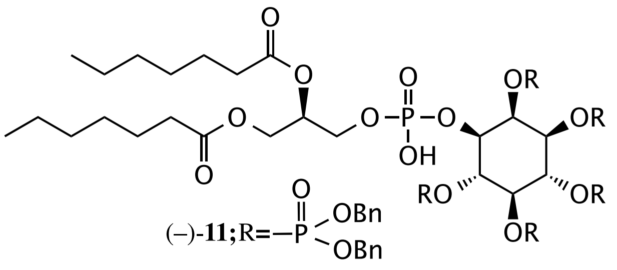
O
O
O-P-O
OH
OR
OR
RO
OR
OR
O
O
O
(–)-11; R=—P
OBn
OBn

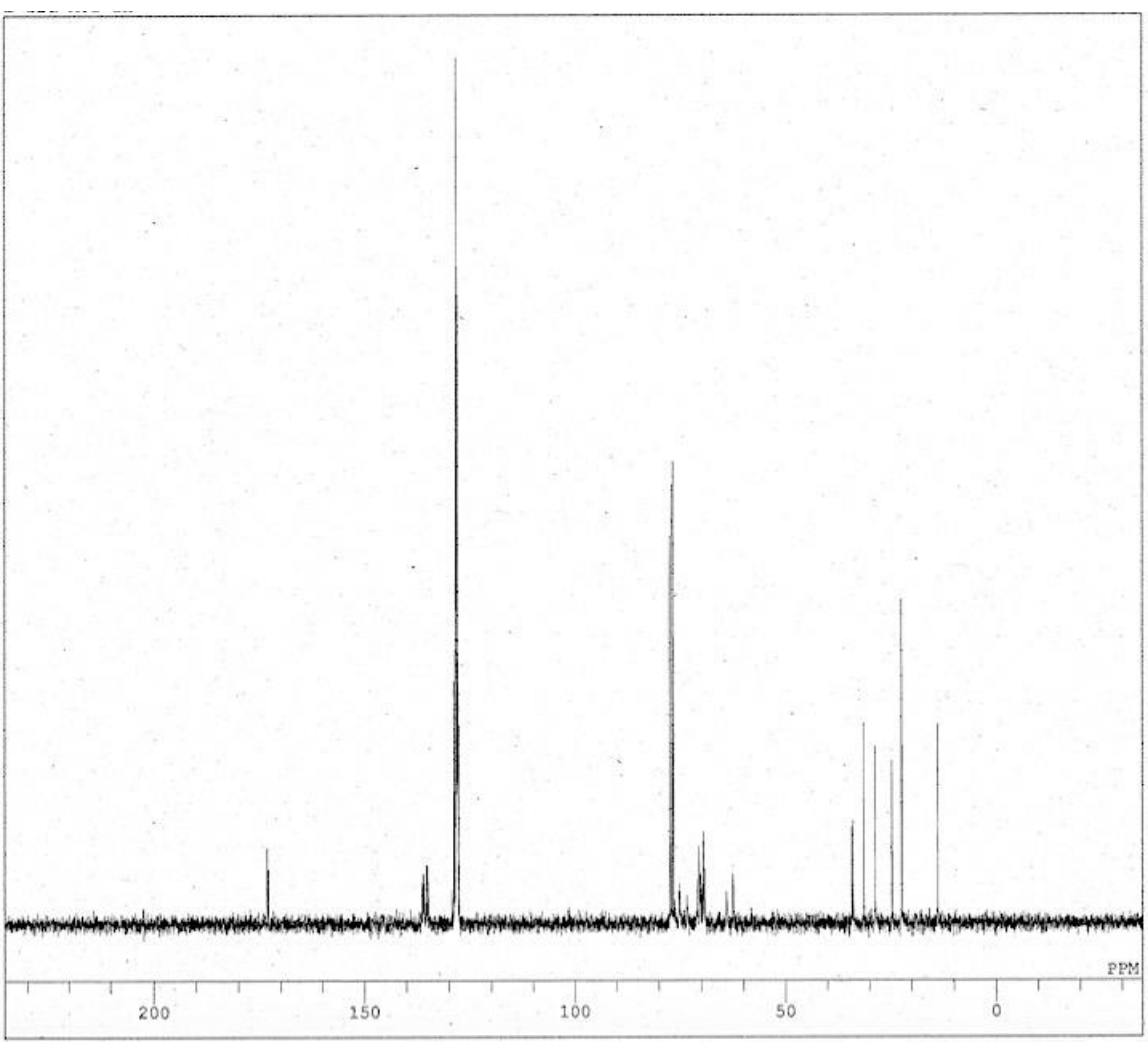
PPM
200
150
100
50
0

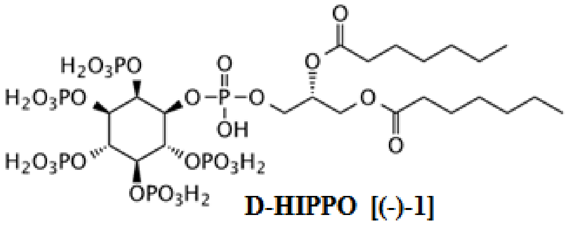

D-HIPPO [(-)-1]

PO_3H_2 = Et_3N^+ salts

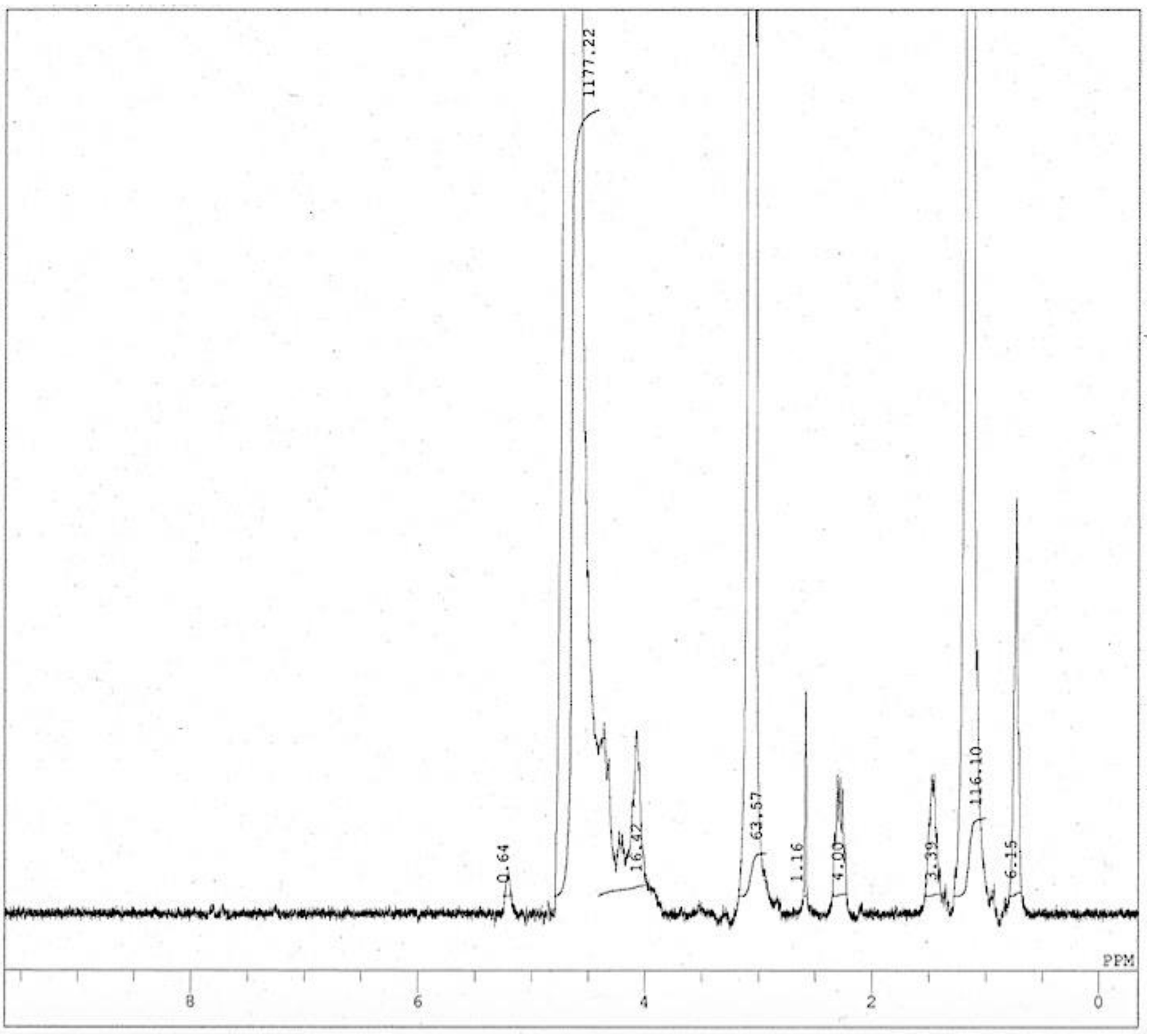

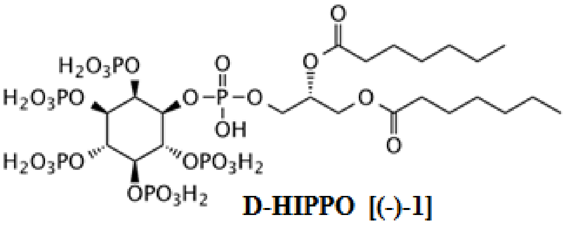

D-HIPPO [(-)-1]

PO_3H_2 = Et_3N^+ salts

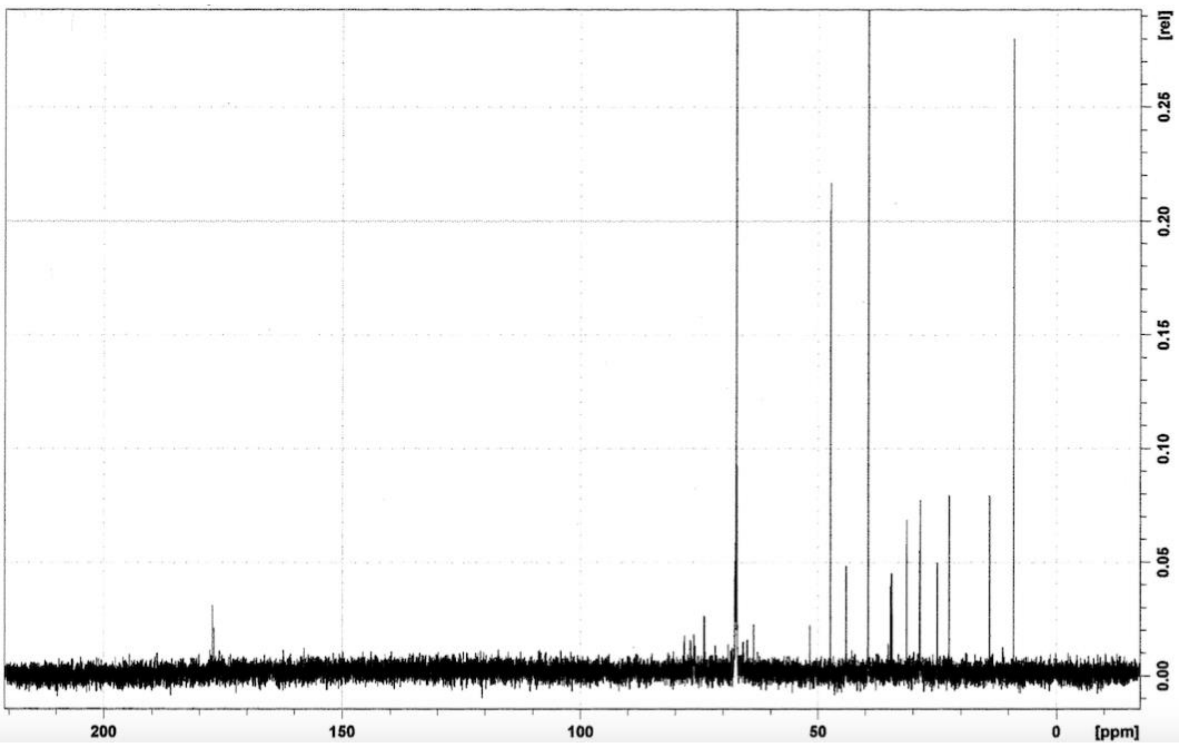

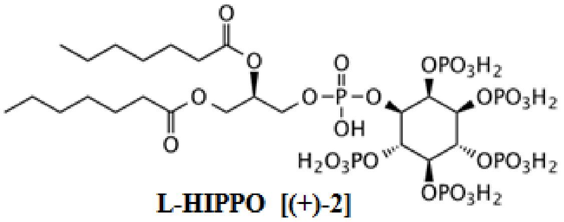

L-HIPPO [(+)-2]
PO_3H_2 = Et_3N^+ salts

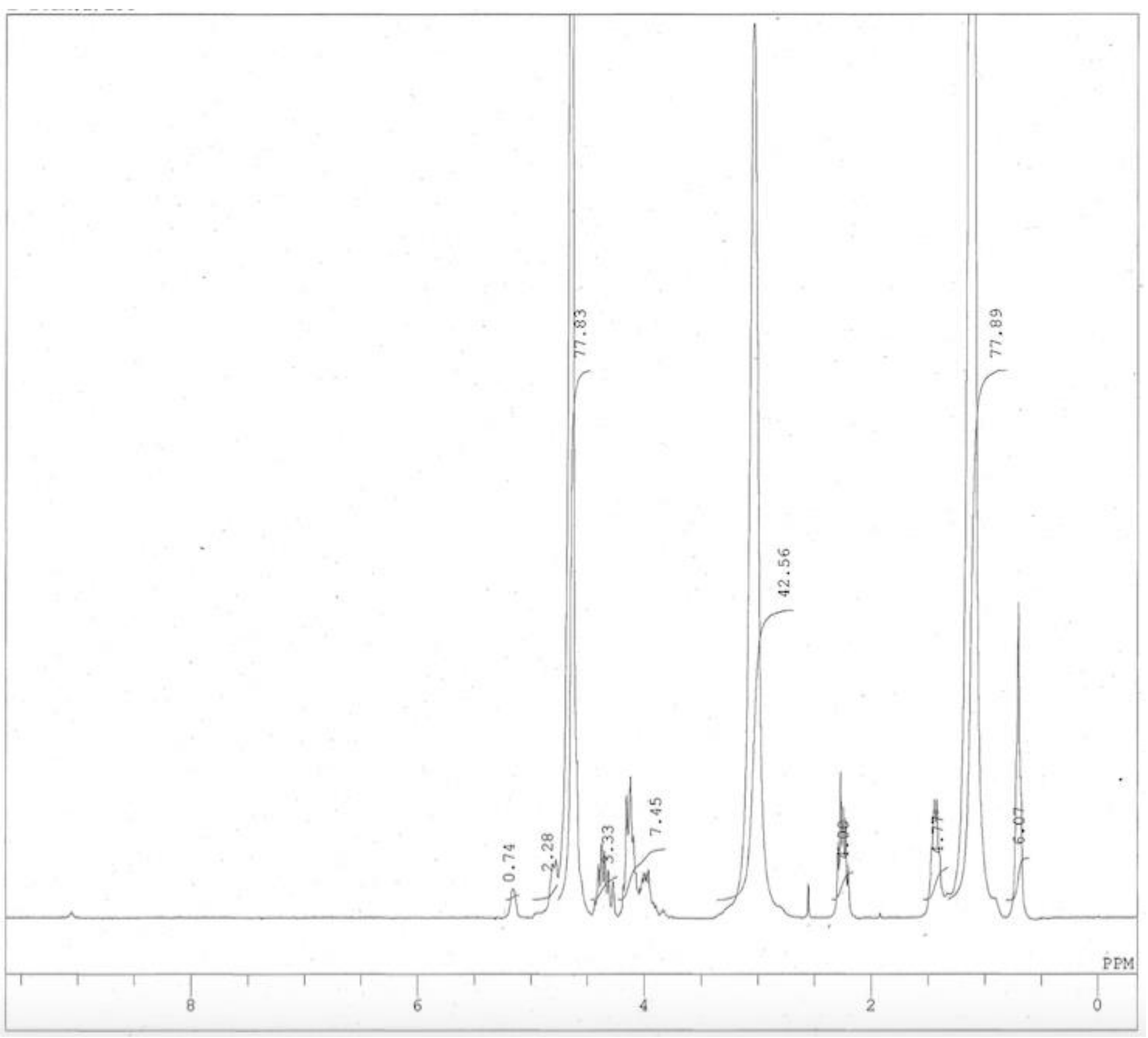

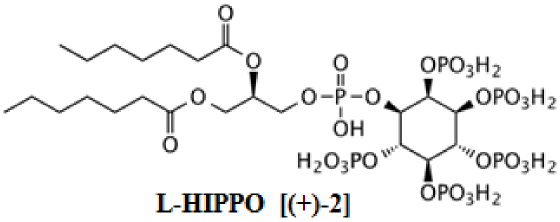

L-HIPPO [(+)-2]
PO_3H_2 = Et_3N^+ salts

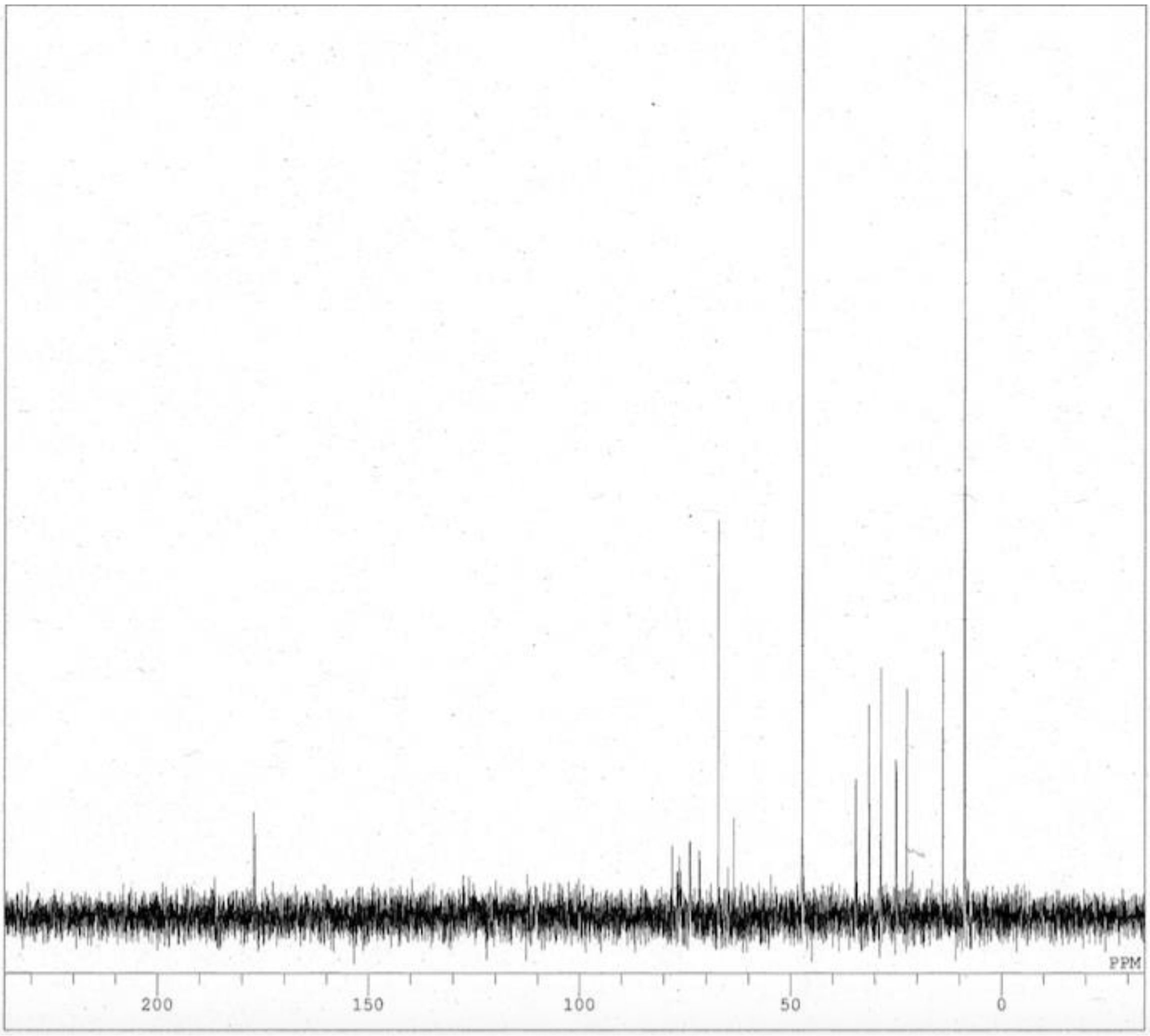

Supplementary Information B:
Binding affinity of D-HIPPO and L-HIPPO to HIV-1 MA domain

Competition assay using surface plasmon resonance (SPR) was performed and dissociation constants (K_d) for MA-binding of the compounds were calculated as previously described[6].

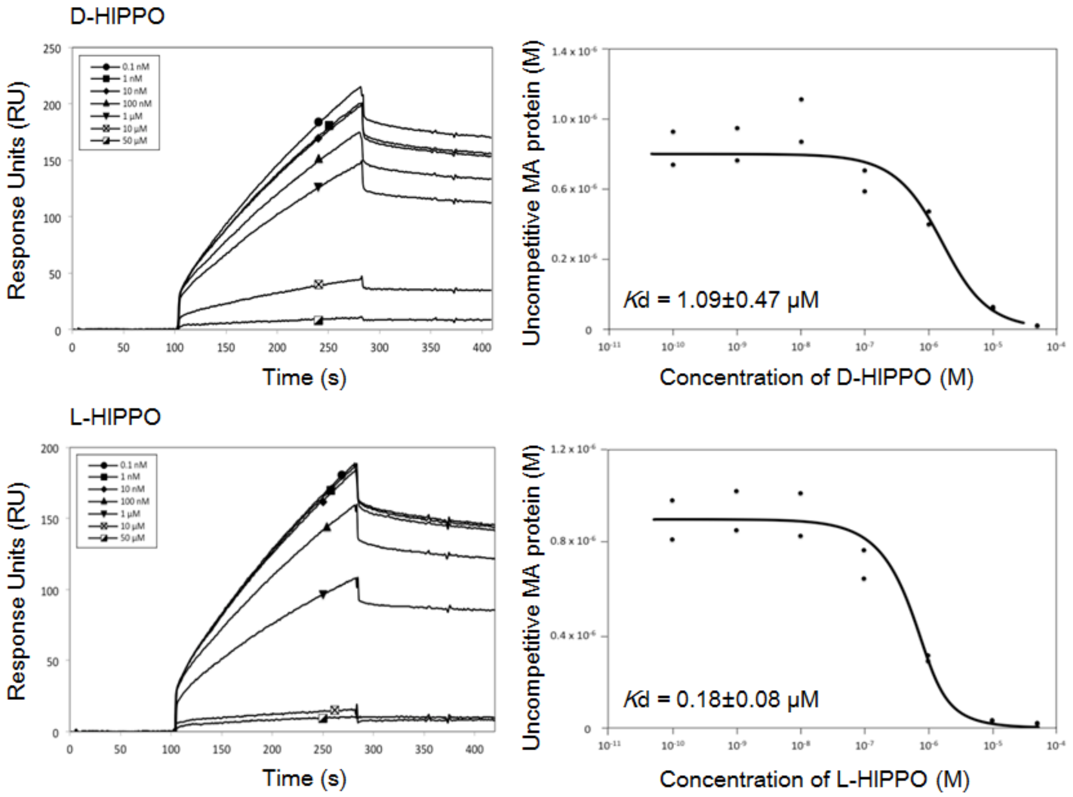

6. Tateishi, H. *et al.* Design and synthesis of lipid-coupled inositol 1,2,3,4,5,6-hexakisphosphate derivatives exhibiting high-affinity binding for the HIV-1 MA domain. *Organic & biomolecular chemistry* **12**, 5006-5022 (2014).

Supplementary Information C:
Death analysis of HeLa cells introduced with L-HIPPO-α-CDE

(a) Cytotoxicity of complex prepared from L-HIPPO (25, 50 or 100 μM) and α-CDE (25 μM) (L-HIPPO-α-CDE) without HIV-1 protein. HeLa cells were treated with the complex, MTT assay was performed at the indicated incubation time. Data from three different experiments are shown as means ± standard deviations. P values were determined using Student’s t test. (b) Apoptotic effect of L-HIPPO-α-CDE without HIV-1 protein. HeLa cells were transfected with pUC19, and after 10 h, L-HIPPO-α-CDE [prepared from L-HIPPO (25 or 50 μM) and α-CDE (25 μM)] was added. After a further 12 h incubation, FACS analysis using annexin V-Cy5 was performed.

(a)

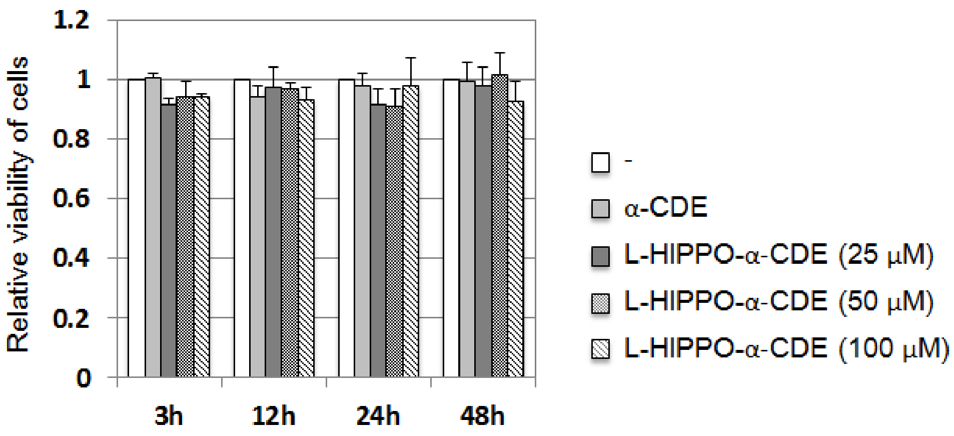

(b) 12h incubation

(1) L-HIPPO-α-CDE (25 μM) (2) L-HIPPO-α-CDE (50 μM)

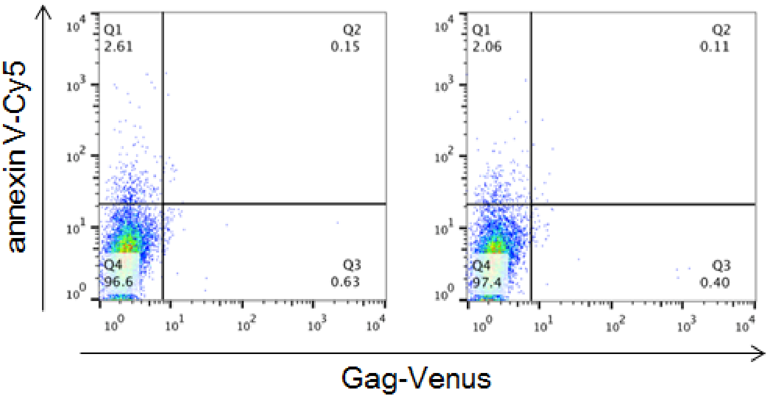

Supplementary Information D:

Bystander effect of cells introduced with L-HIPPO-α-CDE

in the presence of HIV-1 protein

(a) Apoptotic effect of L-HIPPO-α-CDE without HIV-1 protein. HeLa cells were transfected with pUC19 by Lipofectamine 2000 (Life Technologies), and after 10 h, a complex prepared from L-HIPPO (25 μM) and α-CDE (25 μM) (L-HIPPO-α-CDE) was added. After a further 12 h incubation, FACS analysis using annexin V-Cy5 was performed. (b) Apoptotic effect of L-HIPPO-α-CDE with HIV-1 protein. HeLa cells were transfected with pNL4-3/GagVenus by Lipofectamine 2000, and after 10 h, none, α-CDE or L-HIPPO-α-CDE was added. After a further 12 h incubation, FACS analysis using annexin V-Cy5 was performed.

(a)

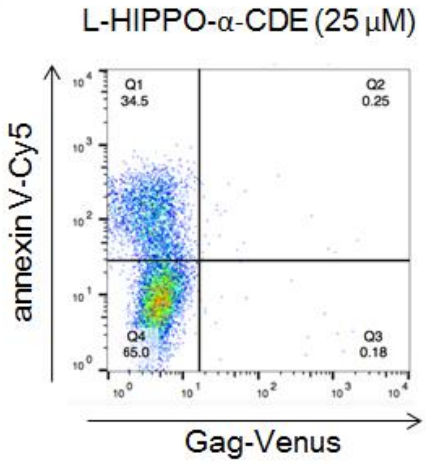

(b)

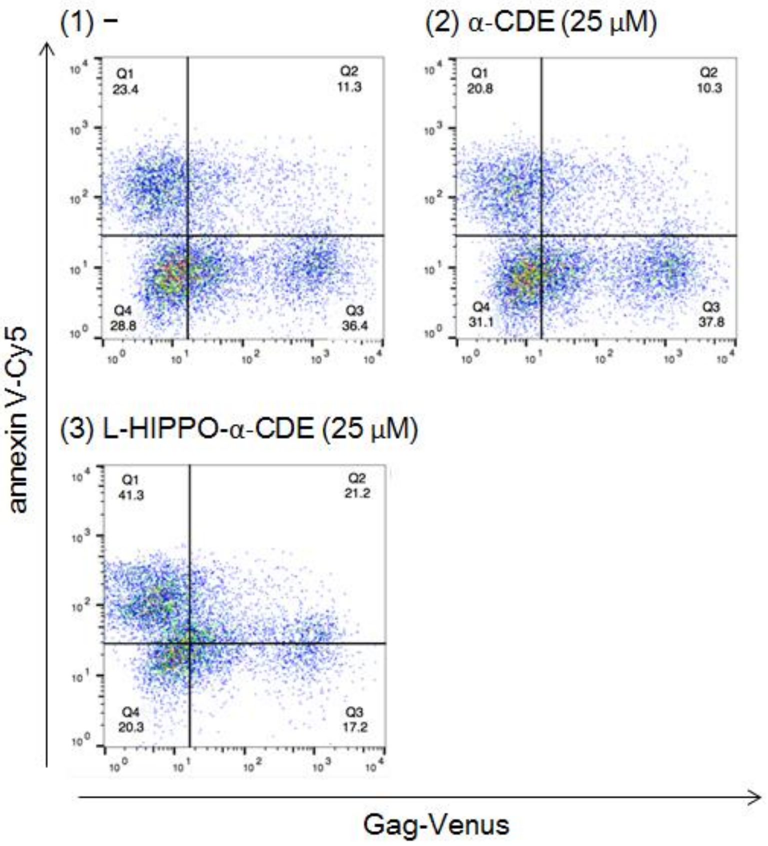
(1) –
(2) α-CDE (25 μM)
(3) L-HIPPO-α-CDE (25 μM)
annexin V-Cy5
Gag-Venus
Q1
23.4
Q2
11.3
Q4
28.8
Q3
36.4
Q1
20.8
Q2
10.3
Q4
31.1
Q3
37.8
Q1
41.3
Q2
21.2
Q4
20.3
Q3
17.2

Supplementary Information E:

Death analysis of Jurkat cells introduced with L-HIPPO-α-CDE

Apoptotic effect of a complex L-HIPPO-α-CDE without HIV-1 protein. To Jurkat cells, a complex prepared from L-HIPPO (100 μM) and α-CDE (25 μM) (L-HIPPO-α-CDE) was added. After a further 12 h incubation, FACS analysis using annexin V-Cy5 was performed.

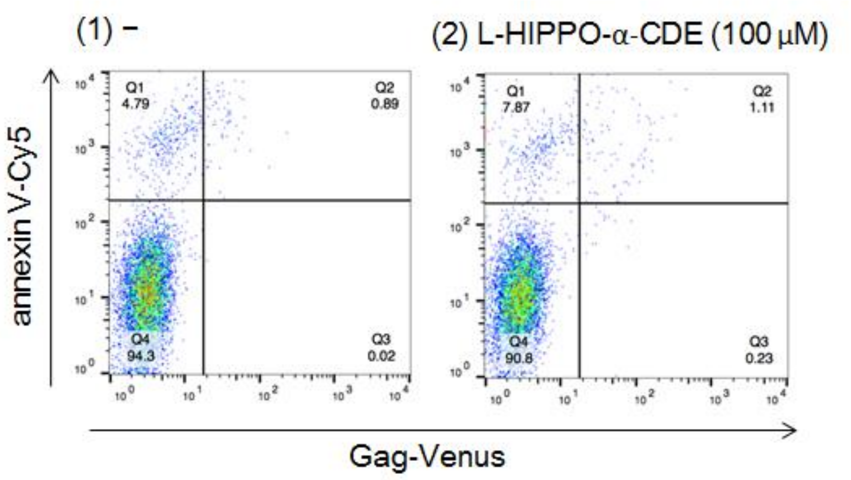

Supplementary Information F:

Wider images of immunoblotting

Full images of “cell” and wider image of “virion” in Figure 2c are shown. In blotting of virion samples, antibody strongly reacted with fetal bovin serum in medium in upper part. Thus only lower part is shown.

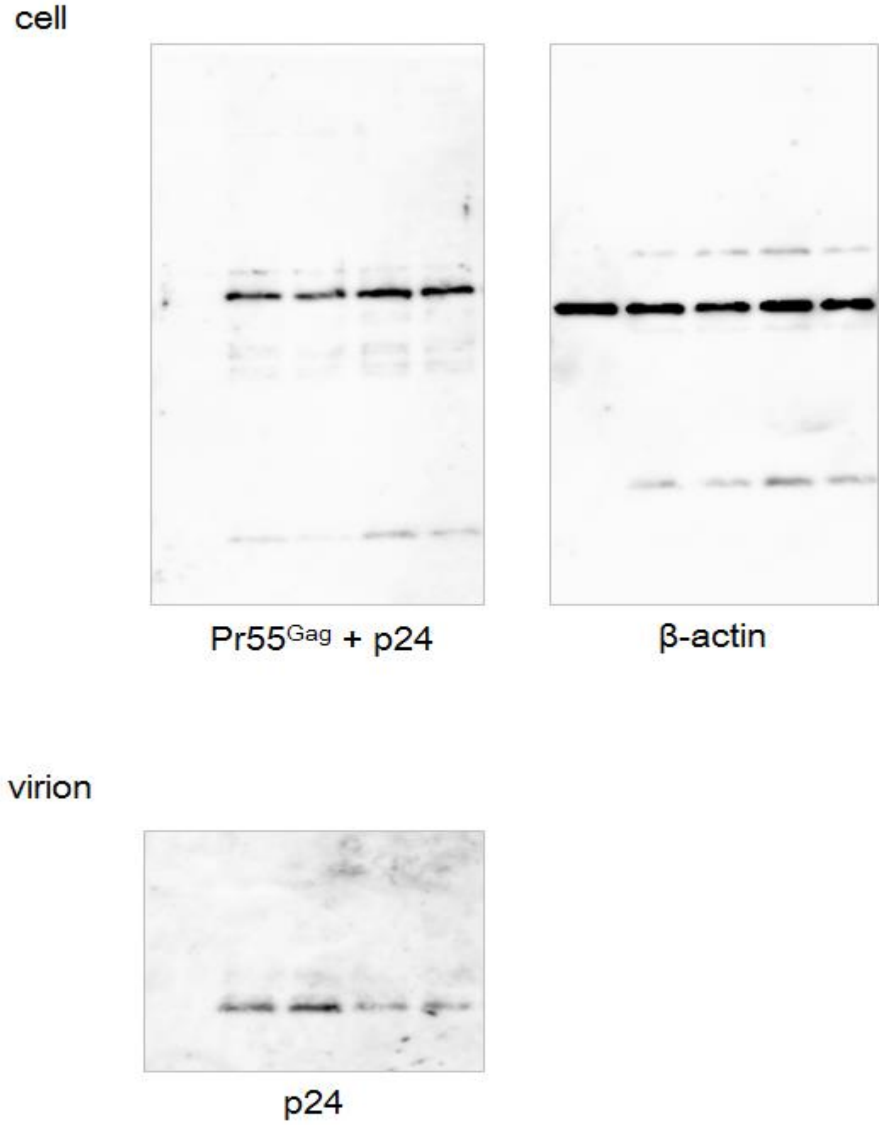

Supplement: Supplementary file 1 — Supplementary Information [file 41598_2017_9129_MOESM1_ESM.pdf]
